# A Descriptive Thematic Review of Barriers, Facilitators, and Recommendations for Cancer Screening Uptake Among Community Dwelling Adults with Mental Ill-Health

**DOI:** 10.3390/ijerph23020216

**Published:** 2026-02-09

**Authors:** Chase Bolton, Sharon Lawn

**Affiliations:** 1 College of Medicine and Public Health, Flinders University, Adelaide, SA 5042, Australia; bolt0104@flinders.edu.au; 2 Lived Experience Australia, Adelaide, SA 5048, Australia

**Keywords:** cancer screening, mental ill-health, barriers, facilitators, recommendations, service users, mental health

## Abstract

**Highlights:**

**Public health relevance—How does this work relate to a public health issue?**

- Cancers and mental health conditions are each among the greatest contributors to the global burden of disease; as comorbid conditions, they pose even greater burdens on individuals, families, healthcare resources, and communities.
- Complexity across the trajectory of cancer screening and treatment for people with comorbid mental ill-health is a significant and multi-faceted public health issue, underpinned by multiple inequalities in the social determinants of health.

**Public health significance—Why is this work of significance to public health?**

- Cancer screening is a pivotal step in addressing cancer burden incidence, mortality, years of life lost, years lived with disability, and disability-adjusted life-years.
- Inequity of access to early cancer screening and treatment is understood as a significant contributor to the up to 20-year gap in life expectancy for people with mental ill-health.

**Public health implications—What are the key implications or messages for practitioners, policy makers and/or researchers in public health?**

- Access to early and regular screening for cancers is vital to address disparities in health status for people with mental health conditions.
- Multiple barriers to cancer screening and treatment at individual, community, health workforce, health service and system levels must be understood and addressed.

**Abstract:**

The much higher rate of premature mortality from cancer among people with mental ill-health is a major contributor to 20-year reduction in life expectancy for this population, relative to the broader population. Under-screening and delays in screening for cancers are recognized as significant issues contributing to this health inequality. This thematic review explored the common barriers to delayed cancer screening, facilitators to overcome those barriers, and the associated recommendations to improve screening rates for people with mental ill-health. Inclusion criteria were peer-reviewed quantitative, qualitative or mixed methods studies and reviews, available in English, involving adults with mental ill-health (experiencing mental distress or with diagnosed mental disorder), living in the community and in contact with primary, secondary or tertiary mental health services, exploring their screening experiences for any type of cancer. The reviewed literature from 37 studies that met the inclusion criteria highlighted key themes contributing to the health disparity experienced by this population, including social determinants of health, comorbidities, and health system factors. Facilitators such as trust, support, self-care, and interventions at a health system level were also highlighted. Study quality was appraised using the MMAT v.18 and CASP tools. All studies that met the inclusion criteria, regardless of quality, were included in the review to provide a comprehensive analysis of the existing literature on this topic. Building upon this literature, further recommendations are presented on how to reduce the cancer screening inequality experienced by people with mental ill-health.

## 1. Introduction

Cancer is among the main causes of death globally, according to the World Health Organization [1]. In 2021, it was reported that, globally, approximately 1.1 billion people were living with diagnosed mental health disorders [2]. The actual number of people living with mental ill-health, of which a broad range of diagnosed mental health disorders (e.g., depression, anxiety disorders, schizophrenia, bipolar disorder) are a subset, is likely to be larger, due to the nature and path of help-seeking, the role of stigma, discrimination and a range of social determinants of mental health [2]. The literature on how mental ill-health is defined is fluid, diverse and sometimes conflicting, depending on where, when and how it is used (e.g., policy documents, clinical contexts, lived experience perspectives, public health perspectives, and so forth). We also recognize that the realities of primary, secondary and tertiary settings in which people have contact for their mental health are also diverse and may use differing terms (e.g., mental illness, mental disorder, mental health problems, mental health conditions, mental health struggles, mental distress, severe mental illness) and that some people who use these services may not yet have a formal mental health diagnosis because this can take time, can change over time, and because determining this may be part of their contact with those services. Therefore, for the purpose of this paper, we use the term ‘mental ill-health’ as an umbrella term to include mental distress (significantly impacting mood, thinking and behaviour) or diagnosed mental health disorders. 

Despite the high prevalence of mental ill-health, significant health inequalities persist for this population, particularly regarding cancer screening [3,4,5,6,7]. The rate of premature mortality from cancer among people with mental ill-health in Australia is approximately 16 per day, in comparison with an expected 1.8 deaths per day in the general population [6]. This contributes to the reported 20-year reduction in life expectancy relative to the general population [3,4,6,8]. Similar concerning disparities between mental health and cancer outcomes have been reported in other countries [9,10,11]. For example, a review of the international literature on depression and anxiety in relation to cancer incidence and mortality [9] found that they were associated with a significantly increased risk of cancer incidence (adjusted RR: 1.13, 95% CI: 1.06–1.19), cancer-specific mortality (1.21, 1.16–1.26), and all-cause mortality in cancer patients, though high clinical and methodological heterogeneity of the 51 included studies was noted. Chiezi et al. [11] state that the findings have been mixed and inclusive for epidemiological studies examining the relationship between common mental disorders (like depression and anxiety).

In the existing international literature, it is well-reported that people with mental ill-health are under-screened or receive delayed cancer screening, thus contributing to a lower life expectancy in this population when compared with the general population [4,6,8,12,13,14]. An international systematic review and meta-analysis of 47 publications, undertaken to determine whether people with mental illness undergo less cancer screening compared with the general population, involved 4,717,839 individuals (501,559 with mental illness, 4,216,280 controls; 69.85% women). They found that screening was significantly less frequent in people with any mental disorder compared with the general population for any cancer, especially for women with schizophrenia [4]. In Australia, Kisely et al. [12], in linking mental health records of Western Australia with cancer registrations and death records from 1 January 1988, to 31 December 2007, sought to investigate the extent to which increased mortality from cancer in psychiatric patients was associated with later cancer presentation and diagnosis (advanced disease), and whether, once diagnosed, there was equity of access to surgery, radiotherapy, and chemotherapy. They found that the proportion of cancer with metastases at presentation was significantly higher in psychiatric patients (7.1%; 95% CI, 6.5–7.8%) than in the general population (6.1%; 95% CI, 6.0–6.2%). These patients were also less likely to have surgery (e.g., for colorectal, breast, and cervical cancers) and less likely to receive radiology (e.g., for breast, colorectal and uterine cancers) and fewer chemotherapy sessions [12].

In Australia in particular, breast and lung cancer are amongst the top cancers that are the leading causes of death, and account for the higher earlier-than-expected death rates, for people with mental ill-health [6,13,14]. For breast cancer specifically, it is reported that individuals with mental ill-health in Australia are six times more likely to die prematurely from the disease than those in the general population [4]. Similarly high rates of earlier-than-expected deaths from breast, lung, and other cancers for people with mental ill-health have been reported in research in other countries, though with some variability [15,16,17,18]. For example, a meta-analysis of data on the mortality risk of breast, colon, lung and prostate cancers for 1,162,971 participants with schizophrenia [15] (four studies in Europe, two in north America and one in Japan), found that people with schizophrenia have a significantly higher risk of mortality from breast, colon, and lung cancer. A UK-wide matched cohort study, investigating the relationship between severe mental illness (SMI) and cancer incidence and mortality [16] involving 69,632 patients, found the rate of all-cancer diagnoses was reduced, especially for people with schizophrenia. More specifically, they found increased mortality for people with SMI with breast cancer or bowel (colon) cancer, especially for people with schizophrenia, but no differences in mortality rate of lung and prostate cancer between people with and without SMI, except for people with schizophrenia (increased mortality rate) [16]. An Italian study involving registry data for 12,385 people with SMI during a 10-year period (2008–2017) (64.1% schizophrenia spectrum and 36.9% bipolar disorders) [17] found that, compared with the general regional population, the mortality for cancer was approximately 50% higher among people with SMI; with the highest mortality rates being those for stomach, central nervous system, lung, and pancreatic cancer. In New Zealand, among 8762 people with breast and 4022 with colorectal cancer, those with schizophrenia, schizoaffective, or bipolar disorders received their cancer diagnosis at a later stage and had two and half times higher mortality for breast cancer and three times for colon cancer than people without SMI [18].

Despite the evident health inequality in uptake of or access to cancer screening, solutions to delayed screening services in these mental health service populations are limited [3]. It is unclear why people with mental ill-health (hereafter referred to as ‘service users’ where applicable) receive delayed screening or are under-screened.

Addressing this health inequality is relevant to health practitioners, people with mental ill-health, and family, carers and kin providing support to them. Identifying factors that contribute to delayed cancer screening can help healthcare practitioners to improve their practices, be more proactive and conscientious in their interactions with service users who experience mental ill-health, and improve their communication with other healthcare practitioners to prevent delayed cancer screening. Similarly, identifying factors that contribute to delayed cancer screening for service users with mental ill-health can shed light on the role that support systems might play in contributing to this issue, and how they can help to address it.

The aim of the review is to map and synthesize existing evidence on barriers, facilitators and recommendations for cancer screening uptake among community dwelling adults with mental ill-health. We do this in three ways: firstly, by identifying themes that contribute to delayed cancer screening in this population; secondly, by identifying themes that facilitate cancer screening in this population; and thirdly, by collecting recommendations and suggesting what can be done to improve cancer screening for people with mental ill-health. Hence, this descriptive review brings the existing evidence together rather than interpret it in-depth.

## 2. Materials and Methods

This study was a thematic review informed by a systematic process under PROSPERO registration number CRD42024496431 (registration date: 27 February 2024).

The study included adults ≥ 18 years of age who were mental health service users, residing in community settings, with contact across primary, secondary, and/or tertiary health systems. In primary care, these are services such as general practices and suicide prevention services; in secondary care, these are services such as public community mental health clinics; and in tertiary care, these are services such as inpatient psychiatric units. Eligible articles were peer-reviewed, published in English, and had a publication timeframe from inception or records to 18 February 2024.

We excluded studies involving adolescents, populations not in community settings (e.g., prisons or other institutions), editorials, conference papers, unpublished theses, reports, protocols, studies not reporting mental health sample data separately, and studies not reporting service user perspectives and/or lived experiences of cancer screening (see Table 1). Barriers, facilitators, and recommendations were not used as inclusion criteria but were explored as outcomes later during the data extraction stage. As this is a broad thematic review, not a systematic review, for included reviews, we have taken the approach of treating each review as its own ‘study’ and summarized information from each review’s overall results, discussion and conclusions.

Assistance from the Flinders University research librarian was utilized in developing the search strategy. MEDLINE, (APA) PsycINFO, Scopus, and EMcare databases were searched via OVID (for MEDLINE, PsycINFO, and EMcare) and Elsevier (for Scopus) with a first search on 2 July 2023 and a final updated search on 18 February 2024. A keyword search was implemented using the following groups of terms: (1) “mental health” OR “mental illness”, (2) “miss*” OR “less likely” OR “reduced” OR “under-screened” OR “influences”, and (3) “cancer screening” OR “cancer prevention” OR “cancer detection”. The terms were then combined (1 and 2 and 3) to identify relevant literature (see Supplementary File S1 Table S1 for a Medline search exemplar). Searches were limited to English-language, peer-reviewed journal articles.

After removing 270 duplicates from the initial 433 records in Covidence, 163 studies were screened and 41 underwent full-text review, resulting in 37 included studies that clearly met the inclusion criteria. Data extraction was piloted with a small number of included studies. The two reviewers (SL and CB) then discussed/checked the parsimony of the piloted approach in capturing the content of interest in answering the research project’s aim before proceeding with extracting data across the identified included papers. The two reviewers (SL and CB) independently screened titles/abstracts and full texts, with discrepancies resolved via discussion. A descriptive summary table, generated using Microsoft Word, was tabulated for comparison amongst the eligible articles. This summary included the surname of first author/year/country, study aims/purpose, participant samples, methods/design, findings/conclusions, and limitations (Supplementary File S2 Table S2). The first reviewer (CB) extracted all of the data and the second reviewer (SL) independently and randomly selected ~20% of the included articles, reviewing them against the data collected by the first reviewer to ensure accuracy and consistency in approach, before reviewing the complete collected summary data to ensure a consistent and succinct format. The reviewers worked independently and resolved any disagreements via discussion. Results of the review were summarized in a PRISMA flow chart to display each review step [19].

Outcomes of interest were barriers, facilitators, and recommendations related to cancer screening among service users. Barriers were defined as factors that prevented or delayed engagement with cancer screening; facilitators were defined as factors that prompted engagement; recommendations were defined as suggestions by articles on how to improve cancer screening uptake. The time-points when data were collected for the eligible articles were conducted heterogeneously. For instance, some studies used retrospective data collection. Similarly, the measurement methods were also done heterogeneously: for instance, some studies were cross-sectional surveys while others were cohort studies.

A deductive thematic framework using predefined outcome domains of interest (barriers, facilitators, and recommendations) as the main themes, was applied to the data. All extracted results that aligned with these outcome domains in each study were explicitly sought. No changes were made to the inclusion or definition of outcome domains, or to the importance given to them in the review. No changes were made to the processes used to select results within eligible outcome domains. Within this deductive structure, inductive subthemes were developed based on emerging patterns from the extracted data within each of the three domains of interest. Facilitators and barriers to cancer screening uptake were considered the most important outcome domains for interpreting the review’s conclusions as they reflected the experiences of mental health service users with screening services. Experiences with screening services have the potential to influence screening rates/uptake.

Study quality was independently assessed by the two reviewers using the Mixed Methods Appraisal Tool Version 2018 (MMAT v.18) for qualitative, quantitative, and mixed methods studies [20], and the Critical Appraisal Skills Programme (CASP) quality rating items for systematic reviews [21]. One reviewer (CB) assessed all of the studies; the second reviewer (SL) randomly screened 20% of the included studies using these quality rating tools prior to finalization of the quality rating tables. Discrepancies were resolved amongst the two reviewers via discussion.

Overall, a majority of included studies were rated as moderate to high quality (3–5 stars) on the MMAT v.18 across the different study designs (see Supplementary File S3 Tables S3–S7 for further detail). We therefore concluded that there was low risk of bias for the extracted data types.

Overall, a majority of the systemic reviews satisfied the CASP quality rating items, being scored as yes (Y) across most or all questions. However, some uncertainty remained, particularly regarding the precision of results and applicability to local populations (see Supplementary File S3 Table S8 for further detail). Across all study types, no processes were used to obtain/confirm relevant further information from original study investigators.

We conducted a thematic analysis of findings based on Braun and Clarke’s six-phase approach [22]. We first reviewed the 37 included studies (Phase 1: familiarization with the data) [22]. To facilitate a thorough examination of the findings, we undertook a series of steps to curate and display the data. Following the initial population of a descriptive summary table, we examined data within the articles specific to our study aims. To achieve this, a summary of findings table was generated to visually display the overall findings of individual articles regarding barriers, facilitators and recommendations for addressing under-screening (Phase 2: generating initial codes) [22]. The summary of findings table included the surname of first author/year/country, aims/purpose, and findings (barriers, facilitators, and recommendations (Supplementary File S4 Table S9)). From the summary of findings table, inductive coding was performed to group codes into broader themes (Phase 3: searching for themes) [22]. To visually display the major themes amongst articles, we generated dedicated thematic tables (Phase 4: reviewing themes), each separately displaying barriers, facilitators and recommendations, with common themes and subthemes noted (Phase 5: defining and naming themes; see Supplementary Files S5–S7 Tables S10–S12) [22]. Finally, phase 6 involved reporting and analysing the themes in this review [22].

Given the heterogeneity of the included studies (related to their methods, populations, contexts and settings) and the gaps in knowledge on this topic, our aim was to bring both depth and breadth to map and synthesize existing evidence on barriers, facilitators and recommendations, so as to provide a more complete picture of that existing evidence so that emerging barriers and innovations are included. Therefore, single study findings were also included within the thematic analysis. Supplementary files S5–S7 Tables S10–S12 provide more detail about the dataset pertaining to barriers, facilitators and recommendations. They provide detailed summaries of the recurring patterns of themes and subthemes (where single study evidence was more apparent).

## 3. Results

**Figure 1:**
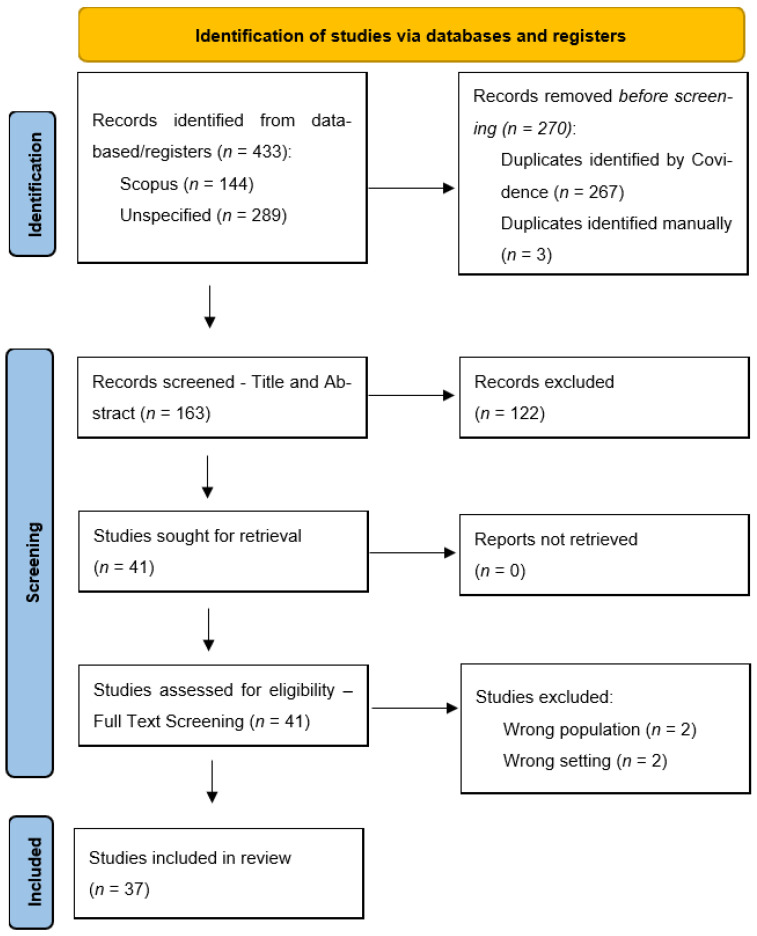
PRISMA flow diagram (Source: [19]).

Of the 37 papers identified in this review (see Figure 1), 20 were conducted in the United States of America (USA), seven in the United Kingdom (UK), 4 in Australia, 2 in Canada, and 1 each in Denmark, the Netherlands, Spain, and Sweden. Nineteen studies focused on cancer screening for women with mental illness, with the remainder involving women and men in their samples. Age of sample populations varied from 18+ years to a focus on older populations. Sample sizes also varied, influenced by the type of study designs, with the cohort studies involving many thousands in their samples. Most studies involved analysis of existing data from specific registries or large administrative datasets or were cross-sectional studies (*n* = 19). In addition to these studies, there were seven reviews, one randomized controlled trial (RCT), five non-randomized controlled trials), two mixed method designs and three qualitative studies.

The type of cancer screening in focus also varied across the studies [23,24,25,26,27,28,29,30,31,32,33,34,35,36,37,38,39,40,41,42,43,44,45,46,47,48,49,50,51,52,53,54,55,56,57,58,59] (see Table 2), with 24 studies focused on specific types of cancer screening. The remaining studies (*n* = 13) involved cancer screening of any type and, more broadly, included some studies with specific groups, including people with comorbid substance abuse disorder and mental illness [47], and military veterans [54,55].

There were three major themes involving barriers to screening: social determinants of health, mental health/comorbidities, and the health system. Each major theme then had various subthemes within them. There were nine subthemes identified within social determinants of health, six within mental health/comorbidities, and five within the health system. These are summarized below [see Supplementary File S5 Table S10 for further detail].

### 3.1. Social Determinants of Health as a Barrier

Social determinants of health are the social and demographic conditions that affect the biological and psychological mental health of individuals’ lives [60].

#### 3.1.1. Education

Low levels of health literacy and education were identified as major barriers for cancer screening due to individuals not fully understanding the associated risks or information health practitioners provided. Individuals with lower levels of education tend to be screened for cancers less than those with higher levels of education/health literacy [30,31,36,37,40,44]. Irwin mentioned that health care providers might not be educating patients about smoking cessation appropriately [44]. However, it appears that there is a significant gap in the literature regarding how health practitioners explain information to people with mental health conditions.

#### 3.1.2. Age

Various literature cited increasing age as another major barrier to cancer screening [23,32,33,36,37,45,48,49]. There seemed to be a negative association between age and cancer screening rates, particularly in cervical and breast cancer screening [37]. Older aged females were more likely to have missed Pap test and mammograms for cervical cancer and breast cancer, respectively [37]. Compared with other females in New South Wales (NSW), women 65 years old or greater with mental illness received 41% less screening and women 34 years or younger with mental illness received 5–10% less screening [33]. Variations amongst age groups might be attributable to the cohort effect [33]. However, one report found that screening in individuals with mental health conditions was low regardless of the age group [24].

#### 3.1.3. Low Income and Finances

Financial costs, lack of insurance, and low income were other barriers abundantly cited in the literature [30,31,33,34,37,41,42,48,49,54,56,57]. One article found this to be particularly problematic for cervical and breast cancer screening [37]; however, another article noted that this is a barrier that many people experience, not just mental health service users [34].

#### 3.1.4. Distance

Lack of transport to enable travel to screening facilities prevented some service users from being able to obtain cancer screening [46,48,49]. Additionally, the further away the screening facility is, the more difficult it can be to physically get there [24,31,33,37]. Furthermore, screening participation was impeded when a screening facility was unfamiliar to the service user; especially for mammography [38]. Unfamiliar screening facilities contributed to lack of familiarity with staff at that location and thus less trust in the facility [38].

#### 3.1.5. Reduced Access

A reduction in access to either health care or cancer screening prevented some service users from being screened in an appropriately timed manner [24,33,34,41,52,56,57]. Several articles found that being isolated from society or from the health system led to reduced screening [23,34,44,57].

#### 3.1.6. Gender and Socio-Economic Status

The gender of the health providers and service users impacted screening participation. Female health providers were preferred by female service users, particularly for cervical screening or history of sexual assault [25,36]. For colorectal cancer screening, non-adherence was greater if the service user was female compared with male [40]. Socioeconomic status was a major sub-theme identified in the literature. In all levels of socioeconomic status, women with mental illness were under-screened for breast cancer [24].

#### 3.1.7. Culture

Culture can impact screening rates in mental health service users, particularly for minority cultural groups whose primary language is different to the dominant language used by services and systems [22,30,31,36]. Tsai found that survivors of breast and prostate cancer were under-screened if they were non-Hispanic other (NHO)/Hispanic, as opposed to other races, including non-Hispanic White (NHW) and non-Hispanic Black (NHB) [42] (pp. E714–E724). Being Aboriginal or Torres Strait Islander also resulted in less screening participation [24].

#### 3.1.8. Never Married

Three articles found that never having married influenced cancer screening rates. There was an association between missing a Pap test and women who had never been married [16]. Never being married possibly results in women having not encountered preventative gynaecological services before [30]. More generally, being single was a barrier to accessing preventative cancer screening [31].

#### 3.1.9. Reproductive History

Reproductive history, especially a prior abortion, negatively impacted cervical cancer screening participation rates [30]. Women with more than four children or who have had an abortion were more likely to not participate in cancer screening [30].

### 3.2. Mental Health or Medical Comorbidities as a Barrier

#### 3.2.1. Mental Health Condition and Severity

The more severe the mental health condition, the more this negatively impacted cancer screening participation [24,32,38]. Woodhead attributed this reduction to people with mental ill-health being less likely to seek help [38] (p. 819). Cervical cancer screening was impacted negatively by anxiety and depression [35], and depression also negatively impacted breast cancer screening [37]. Additionally, depressive symptoms might enhance the feeling of helplessness and then further deter individuals from seeking help [36]. Vigod, however, found that, even with more appointments to see a primary care physician, mental health service users still received less cancer screening [39] (pp. 159–168). However, increased number of hospital admissions negatively influenced screening adherence [23].

Some literature report that having a mental health condition results in decreased screening for service users [26,45,56]. Furthermore, the severity of the mental health condition influenced participation in cancer screening [45], and some service users under-estimated their risk of cancer [44]. It seems like the more psychological distress the service user is experiencing, the less likely they are screened for cancer [39]. Some literature found mixed results depending on the type of mental health diagnosis [23,27,36,50].

#### 3.2.2. Medical (Non-Psychiatric) Comorbidities

There seems to be a negative association between poor physical and/or mental health and cancer screening [30,36]; generally, the poorer one’s physical or mental health, the lower the screening rate [42]. Aggarwal has suggested that comorbidities have the potential to distract the health provider from cancer screening [36] (pp. 392–398).

#### 3.2.3. Cognitive Difficulties/Impairment and Motivation

Mental health service users may experience cognitive difficulties and/or impairment that then impacts their participation in preventative care services such as cancer screening [29,30,44,52,56,58]. Mental health conditions can negatively affect individuals’ motivation and concentration, which then prevents them from accessing screening services [23,29,38,42,46,52]. This can include problems with recall, which prveents them from actioning referrals for screening and attending follow-up appointments.

#### 3.2.4. Embarrassment/Stigma/Shame

Embarrassment, stigma, and shame surrounding mental health has been suggested to be a common potential barrier to cancer screning [25,33,34,35,48,49,56,58], specifically for breast and cervical cancer screening [24]. This stigma results in reduced access to care [57]. The prejudiced attitudes of health practitoners also contribute to the barrier for mental health service users when accessing screening [51]. Interestingly, one article found that stigma was a barrier identified by both service users and mental health professionals [46]. Another article identified that embarassment was a particular barrier for obese women [30].

#### 3.2.5. Patient Concerns About Screening (e.g., Trauma, Procedures, Fear)

Fear or distrust due to past trauma experiences as part of receiving psychiatric treatment, and associated concerns with the screening process itself, have been noted as key barriers to cancer screening [25,33,34,57]. Some women tend to delay/avoid breast and cervical cancers specifically if they had a history of sexual trauma [24]. Other service users reported that they were under-screened because they feared receiving bad news and because of the potential for trauma [46].

Two articles noted that the nature of the screening test might be a factor that deters service users from participating in screening; specifically for cervical screening. Cervical cacner screening is an “intimate” process that service users might not feel comfortable with [33]. Some service users also delay/avoid getting cervical screening because the process feels “impersonal” to them [31].

#### 3.2.6. Smoking

Smoking was found to be a barrier to cancer screening in service users with mental illness. One article attributed the decrease in screening participation to smokers already engaging in one unhealthy activity (smoking) and being more prone to participate in other unhealthy behaviours [30]. People with mental ill-health have a higher chance of engaging in unhealthy activities such as smoking and being under-screened for cancer [41]. More generally, Zhang found that service users who smoked were less likely to receive their cervical and breast cancer screening [37].

### 3.3. The Health System as a Barrier

#### 3.3.1. Delaying Care/Prioritization

Many service users were under-screened for cancer because either they or their healthcare provider delayed or placed it as a lower priority when compared with other medical conditions or compared with their mental health conditions [25,35,40,57,58]. Specifically, breast and cervical cancer screening were delayed if service users experienced symptoms such as anxiety or if they did not prioritize timely screening [37].

Lack of time is a barrier that is shared amongst mental health service users, the broader population, and health professionals [34,46]. Health professionals might not have enough time to screen individuals, while service users have difficulty dedicating a block of their time to get screened [46].

#### 3.3.2. No Primary Care Provider

Not having a primary care provider has typically been associated with reduced cancer screening, though the reasons for this vary. This included simply not having one [35,48,49,56], problems with the availability of primary care providers [54], or service user avoidance of the primary care setting [51]. One article found that not having a primary care provider was a mixed facilitator and barrier to cancer screening [36].

#### 3.3.3. Siloed Health Systems

Lack of integration amongst mental health service users and physical health services was found to be a barrier to screening participation [44,58]. System siloes include a lack of connection between cancer screening services and community mental health facilities [54] and lack of follow-up within and between services [34]. Siloes between systems were a barrier also encountered by the general population [34]. For health practitioners within these siloed systems, ambiguity or lack of role definition also resulted in under-screening service users for cancer [23,44]; that is, if responsibility for cancer screening was unclear, no one took responsibility.

Lack of communicaiton within siloed systems of care was found to be a common barrier in the literature. Reduced communication amongst psychiatrists and primary care physicians was noted to be a barrier to cancer screening [48,49]. Similarly, lack of communication between various professionals in the health system contributed to lower screening rates, perhaps due to the uneven distribution of training for workers to develop their communciation skills [46].

The effect of lack of integration seemed to be particularly evident for breast and cervical cancer screening [24]. One article found a lack of integration to be a mixed barrier/facilitator according to the type of mental health condition, and that the integration of mental health treatment in primary care practices might explain why patients with major depressive disorder in medical homes used more preventive services compared with those with mental health conditions [50]. A lack of reminders seems to be associated with lower levels of screening participation [34,48,49]. Mkuu noted that this barrier is experienced by both mental health service users and the braoder population [34] (p. 252).

#### 3.3.4. Physician Knowledge/Identification of Screening Candidates

Physicians’ lack of knowledge pertaining to mental illness and/or screening procedures serve as barriers to screening [46], as does the lack of knowledge of psychiatric staff in terms of cancer screening [23]. Baillargeon discovered that an incomplete health evaulation might be a barrier for receiving appropriate screening [41]. They noted that mental health service users might “drop out” prior to the health practitioner completing the cancer staging process [41].

#### 3.3.5. Negative Attitudes and Diagnostic Overshadowing

Providers’ negative attitudes towards mental illness are perceived to be a common barrier to cancer screening [24,35]. Additionally, primary care providers that failed to acknowledge mental health complaints made by service users have contributed to lower screening participation [29]. Some health professionals failed to properly identify women that were due for cancer screening, thus resulting in lower participation rates [34]. Similarly, diagnostic overshadowing, where service users’ physical symptoms were dismissed by physicians, was a common barrier cited in the literature [45,46,53].

In terms of facilitators, there were five major themes identified in this review: social determinants of health, increasing uptake in the health system, trust, support, and self-care. Each major theme then had various subthemes within them. There were six subthemes identified within increasing uptake in the health system, two within trust, three within self-care, three within social determinants of health, and two within support. These are summarized below (see Supplementary File S6 Table S11 for further detail).

### 3.4. Social Determinants of Health as a Facilitator

Having the financial means or insurance to afford cancer screening was found to be associated with improved screening rates [26,33,35,40]. Similarly, having access to transport was associated with increased screening rates in service users [25]. Furthermore, holding the screening process at a familiar location led to an associated increase in screening participation amongst service users [46].

### 3.5. Increasing Uptake in the Health System as a Facilitator

Participation and/or cancer screening increase when staff are knowledgeable about the process and confident of their role. Additionally, the implementation of methods such as reminder phone calls, financial incentives, and targeted invitations also increased participation rates. In a similar manner, making the screening process easier for service users via more convenient testing, the use of community-based cancer navigators to aid service users, or the integration of mental and physical healthcare systems also improved screening rates and/or participation.

#### 3.5.1. Knowledge

When staff are aware and knowledgeable about mental illness, service users are more comfortable obtaining healthcare (such as cancer screening) from them [46]. Knowledgeable staff can make the processes easier for service users by recognizing that they have a mental health condition (e.g., more time given for identification, the creation of space for staff to ask and the person to disclose).

#### 3.5.2. Professional Role and Identity

Screening rates were facilitated by mental health professionals who are conscious of their role in promoting screening in service users [46].

#### 3.5.3. Participation, Targeted Invitations, Phone Counselling

Action to implement routine systemic methods to increase participation was determined to be a facilitator in cancer screening uptake rates according to several articles. Reminders, such as phone calls, could serve as prompts for service users [25,46,59]. Targeted invitation and phone counselling were also methods that were found to improve the screening rates amongst service users [35]. Additionally, incentivizing service users (e.g., with a financial incentive) was found to be associated with higher rates of screening uptake [25,38].

#### 3.5.4. Test Convenience

More convenient screening processes were found to be associated with increased screening uptake, particularly for colorectal and cervical cancers. The ability to obtain a self-collected cervical cancer sample increased the convenience of the screening process [33]. In a similar manner, the faecal immunochemical test (FIT) was determined to be more convenient than the guaiac faecal occult blood test (gFOBT) for service users [59].

#### 3.5.5. Community-Based Cancer Navigators

Community-based cancer navigators faciliate cancer screening by working directly with service users to help overcome barriers, link them with services, fill gaps in communication, and to facilitate information exchange and accessibility [53].

#### 3.5.6. Service Integration

Integration of services through the sharing of care pathways or the colocation of services was found to be associated with increased screening rates [25,54].

### 3.6. Trust in the Service and Health Professionals as a Facilitator

Previous positive experiences with the health system, being able to choose the gender of healthcare provider, and having a regular primary care provider all positively impact engagement with cancer screening.

#### 3.6.1. Trust and Positive Experience

When individuals with mental illness trust their primary care provider, this has the potential to lead to improved screening participation [29,36]. Previously having a positive experience with the health system and/or health professionals was found to be a facilitator for screening uptake [25,46]. Having a good prior healthcare experience suggests that individuals would be more likely to return for follow-up care, and also future opportunities for prevention and earlier intervention screening.

#### 3.6.2. Healthcare Provider Gender

Interestingly, provider gender was found to serve as both a barrier and facilitator [36]. If a male practitioner advised an individual to get screened for cervical cancer, it was a barrier; however, if the mammography technician was a female, then this increased screening participation [36].

### 3.7. Presence and Nature of Support as a Facilitator

#### 3.7.1. Support

A solid support network was found to be associated with increased likelihood of partaking in cancer screening; whether it be support from professionals such as the primary care provider or informal social, emotional, or instrumental support such as from family or friends [25,29,36,56]. Perhaps this is because support involves encouragement to be proactive about cancer screening and address the individual’s concerns/fears, practical support to overcome barriers and obtain screening services, and role models for information and advice.

#### 3.7.2. Presence of a Primary Care Provider and Continuity of Care

Most articles identified having a clearly designated primary care provider as a facilitator to cancer screening [29,38,56] because it likely correlated to better continuity of care [35,46,53]. However, one article identified having a primary care provider as a mixed barrier and facilitator depending on the literature [36]. Continuity of care seemed to be particularly beneficial in improving uptake for cervical cancer screening via the Pap test [31].

### 3.8. Positive Approach to Self-Care as a Facilitator

#### 3.8.1. Self-Care

Maintaining one’s self-care was found to be a facilitator for cancer screening [25]. When individuals had poor self-rated health, this tended to facilitate screening participation [40]. Similarly, individuals who were “health conscious” adhered more to cancer screening [46].

#### 3.8.2. Awareness of Physical Symptoms

Being able to identify physical symptoms (e.g., finding a breast lump, which prompted seeking a healthcare check) was found to be associated with increased participation in cancer screening [46].

#### 3.8.3. Diagnosis and Mental Health

Three articles found that having a diagnosis/mental health condition was associated with increased screening [24,30,46]. However, two articles found that the type of diagnosis/mental illness determined whether it was a barrier or facilitator to cancer screening [36,50]. Domino found that service users with less severe mental health conditions (like major depressive disorder) had a lower amount of specialty mental health visits but accessed preventative services in medical homes more than service users with illnesses such as schizophrenia [50]. Aggarwal cited one study that mentioned reduced breast cancer screening with psychotic disorders and another that mentioned increased breast cancer screening with substance use disorders [36].

### 3.9. Recommendations

There were five general recommendations in the literature on how to improve cancer screening for people with mental ill-health: improving the health system to be more responsive to the needs of people with mental ill-health, attention given to the social determinants of health (particularly finances, discrimination, and education), the implementation of strategies to improve patient compliance/participation, further research to understand the experiences of service users, and other/miscellaneous strategies (see Table 3) [see Supplementary File S7 Table S12 for further detail].

#### 3.9.1. Improve Health System

The reviewed studies emphasized the importance of tailored interventions that could increase cancer screening uptake by targeting specific groups and risk factors, or by addressing specific areas of concern. There were some suggestions in the literature on who the targeted groups might include: people with depression and anxiety might benefit from targeted programs [37]; the homeless could be targeted in an attempt to broaden the number of individuals with schizophrenia [44,53]; and minority groups that may be disadvantaged [30]. Interventions that target risk factors for cancers such as lung cancer (e.g., interventions assisting with smoking cessation) were recommended [25,44,53]. Addressing the risk factors of cancer has the potential to make service users more aware of their risks and thus could result in increased screening rates. Similarly, interventions tailored to different age groups could be beneficial [44].

Various literature recommended addressing the apparent lack of integration amongst the physical and mental health systems. Better system integration amongst mental health service providers and primary care/physical health physicians was suggested to enhance screening rates [27,28,44,46,52,53,57,58]. Interventions, such as combining mental health services/providers with physical health services/providers and motivating service users to be more aware of their self-health, were also recommended to be implemented to increase screening uptake [45,58]. Integration of health systems could lead to more holistic care of patients, thus resulting in more cancer screening.

The literature recommended to address barriers to access at various levels, including service user level [52] and physician level (e.g., the need for shared decision making in cancer screening, distinguishing which providers should advise on smoking cessation, and providers’ need for education on smoking cessation in mentally ill-health populations), which could lead to improved screening rates [44]. It is important to consider that mental illness itself can serve as a contributing risk factor in the experience of barriers to care [45]. Strategies need to be developed to consider barriers such as cognitive impairment and psychosocial factors [29]. Additionally, new strategies need to be considered for individuals with complex medication history such as co-occurring chronic medical conditions [54].

Current care models need to be revised to better suit service users and increase screening. It has been recommended that new models of care be investigated and that care plans be executed [53]. Care models could involve co-location [54,56] and referencing ideas from the Quality in the Continuum for Cancer Care framework [45].

#### 3.9.2. Social Determinants of Health

Recommendations regarding social determinants of health, such as finances, discrimination, and education, were proposed as a means of increasing screening rates. If increased funding was advocated for by mental health nurses, service users, and organizations, then this new financial capability could work to enhance patients’ health via resources and education [52]. Assisting service users to understand insurance schemes and how to use their insurance could facilitate cancer screening [34]. Methods to make cancer screening more inclusive included addressing cultural safety [24] and racial/ethnic disparities [42] and catering to different languages [30]. Furthermore, implementation of patient navigation programs was a specific recommendation made to make cancer screening more culturally accessible [42,47].

Providers and service users could benefit from further education. This would involve recommendations for educating providers on potential biases [58] and details about the screening process [44,58], and educating patients on cancer risk factors and preventative measures [32,44] and the cancer screening process [23]. Furthermore, education encouraging shared decision making and implementing trauma-informed care would benefit service users and providers.

#### 3.9.3. Improving Participation

There were some different recommendations in the literature on how to improve compliance and participation. Primary care physicians could directly ask patients about screening compliance [39]. At family physician practices, all staff members could try prompting patients to get screened [35]. Psychiatric professionals could encourage uptake of cervical cancer screening [31]. Perhaps a direct approach would allow for less ambiguity around the screening process and eligibility.

Participation could increase through changes to the screening invitation process. The literature recommended targeting patients that have become lost to follow-up [53], as well as using more user-friendly methods of scheduling appointments [23], and implementing follow-up calls to patients [29]. Addressing issues in the screening invitation process could increase the number of service users that accept the invitation to be screened; thus, increasing screening rates.

A recommendation to reduce the stigma around mental illness was listed in various literature but not further elaborated upon [27,51]. This raises the issue of how stigma pertaining to mental illness could be addressed in the health system.

Communication amongst patients and health professionals (both mental health professionals and primary care providers) could be improved [29]. Health professionals could improve their communication skills with patients by offering specific sessions devoted to delivering results [28] and by improving communication between oncology professionals and mental health professionals [53]. Better communication amongst health professionals and patients could result in a more integrated and patient-focused system that raises awareness about a lack of cancer screening.

Having a good support system is a recommendation made by several articles. Women need to have a social support system to be prompted to participate in screening for breast cancer [29]. General practitioners and mental health professionals can work to support service users [43]. A similar recommendation was made, in which peer support workers could enhance the support network of service users [56]. Support can also be established so that service users feel more comfortable in being able to self-manage [45]. When patients feel well supported (e.g., by peer support workers, social support networks) perhaps they feel more comfortable accessing the health system and feel more at ease undergoing screening procedures.

#### 3.9.4. Research

There are a vast number of recommendations in the literature for additional research to be done. Some recommendations were specific; for instance, research to investigate how to overcome barriers and with regard to whether individuals with severe mental illness have barriers that are solely relevant to them [48,49]. Other recommendations were more generic, for instance more research into barriers and facilitators [26], barriers to cancer screening [35], and research pertaining to contributing processes related to breast cancer [27]. It would also be beneficial to research the impact of mental illness on screening rates (e.g., anxiety and cervical cancer screening) [37]; that is, whether screening rates improve when anxiety or depressive symptoms are treated [39].

Future research focusing on interventions and care settings was also recommended as beneficial. This included the use of community mental health clinics (CMHCs) for cancer screening [44], studies involving a variety of care settings [47], and research into how patient care is affected by primary care-based medical homes [50]. Future research into how individuals with comorbid mental health conditions can obtain better delivery of health services was also recommended [56]. Understanding the impact of various settings and comorbidities can enhance our knowledge of contributing factors to low screening rates amongst service users and identify areas of concern that need to be targeted.

#### 3.9.5. Other/Miscellaneous

It was recommended that strategies need to be implemented at individual, policy, and system levels [28,46]. Multiple studies recommended the need to create clinical guidelines and policies specifically for mental health populations, suggesting that there is currently a clear gap in the health system.

## 4. Discussion

Current literature reports various barriers and facilitators to cancer screening in people with mental ill-health. Common barriers include social determinants of health (e.g., culture, distance, access, finances), mental health/comorbidities, and the health system (e.g., delayed care, siloed health systems, lack of primary care provider). Common facilitators included social determinants of health (e.g., finances, transport, familiar location), increased uptake (e.g., knowledge, participation, targeted invitations, system integration), trust in service and health providers, presence of support, and a positive approach to self-care. Recommendations included improving the health system to be more responsive to the needs of people with mental ill-health, addressing the social determinants of health (particularly finances, discrimination, and education), implementing strategies to improve patient compliance/participation, further research to understand the experiences of service users, and other/miscellaneous strategies.

### 4.1. Alignment with Broader Public Health Frameworks

Overall, our findings seem to align with current policies and guidelines in various countries around the world. The Australian Cancer Plan focuses on improving participation rates in cancer screening programs, addressing social determinants of health (e.g., education, transport, socioeconomic status), addressing modifiable risk factors (e.g., smoking), and implementing targeted, culturally safe screening programs [49]. Other aspects of the Australian Cancer Plan include enhancing trust in the system, utilizing person-centred navigation models of care, integration at profession and health system levels, and reducing racism [61].

The Royal Australian College of General Practitioners (RACGP) published an article that suggests how cancer screening can be improved, particularly in a general practitioner (GP) setting [62]. It mentioned addressing time constraints, providing appropriate support, funding, and training for GPs, further research into the needs of underserviced populations, and the use of liaison oncology services to improve communication in the health system [62]. GPs have a crucial role in the continuity of care and in cancer screening; I believe this article does a good job at highlighting the health disparity that people with mental ill-health experience and serves as a good starting point for how to work towards eliminating this gap from a GP setting.

In the UK, the goals of the NHS Long Term Plan [63] include addressing modifiable risk factors (e.g., tobacco control plan, alcohol, human papilloma virus (HPV) primary screening for cervical cancer) and implementing models of care that are based on evidence [63]. A report from Public Health England further emphasized the disparity in terms of people with severe mental illness being under-screened for cancers and being less likely to participate in cervical, breast, and bowel cancer screening [64].

In the USA, the goals of the National Cancer Plan include optimization of the workforce, increased engagement, addressing stigma regarding cancer screening processes, and the implementation of National breast and cervical cancer early detection programs for those with financial difficulties [65].

In Canada, the goals of the Canadian Strategy for Cancer Control include implementing a national lung cancer screening program, implementing new models of care, having services address the needs of people who are underserviced, improving resources for remote/rural areas, integration, enhanced communication, patient navigators, encouraging smoking cessation and HPV vaccinations, further research into the barrier of minority groups, education aimed to lower the prevalence of racism, and involvement of First Nations, Inuit and Metis communities [66].

### 4.2. Limitations

One limitation of this review is that the keyword search, time period, and selected databases could have resulted in us missing out on relevant studies. A second limitation is that the inductive coding was subject to the authors’ interpretation, thus allowing for potential bias. A third limitation is with regard to how reviews were treated in our thematic analysis: we did not describe or analyse each individual study within each identified review. Therefore, more nuanced information about barriers, facilitators and recommendations may have been overlooked. A final limitation regards the generalizability of the results, as the included studies were from various countries across the world that are likely to have differing health systems. There are likely to be variations in cancer screening recommendations at different levels (e.g., between different countries and organizations within countries). Additionally, only some cancers have screening guidelines for people at average risk, and these guidelines may include restrictions on age—guidelines are likely limited for people at higher risk.

## 5. Conclusions

This study investigated the common barriers and facilitators to cancer screening amongst service users and interventions to improve participation. Key barriers included social determinants of health (e.g., finances, access, distance), the presence of mental health/comorbidities, and aspects of the health system. Key facilitators included social determinants of health (e.g., finance, transport, familiar location), interventions to increase uptake and knowledge, trust, support, and self-care.

Our findings demonstrate that barriers and facilitators are multifactorial and present across personal, professional, and systemic levels. These results emphasize the importance of implementing recommendations to assist with reducing cancer screening barriers experienced by service users. With less delayed cancer screening/under-screening, the drastically higher mortality rates for service users compared with the general population can hopefully be reduced and become more equitable.

The persistent gap in cancer screening for people with mental ill-health highlights the need for further research into the experiences of mental health service users. Understanding their lived experiences may provide insights into why efforts to combat known barriers have not worked to reduce this disparity. Utilizing the lived experiences of service users, future policies and guidelines can be guided to better assist in closing this gap by addressing their needs. In addition, interventions to improve communication between health professionals and service users may make service users feel more supported, facilitating greater participation in cancer screening. Finally, working to eliminate siloed health systems can promote an integrated health system that provides better patient-centred care and results in equitable cancer screening for service users.

## Figures and Tables

**Table 1:** Table of inclusion and exclusion criteria.

Element	Description	Inclusion Criteria	Exclusion Criteria
Population	Adults who are mental health service users.	-Adults ≥ 18 years of age who were mental health service users. -Community dwelling populations.	-Adolescents under 18 years of age. -Populations not in community settings (e.g., prisons).
Concept	Perspectives of mental health service users.	-Studies reporting mental health data separately. -Studies reporting service user perspectives and/or lived experiences of cancer screening.	-Studies not reporting mental health data separately. -Studies not reporting service user perspectives and/or lived experiences of cancer screening.
Context	Health system and community settings.	-Community-based settings with primary/secondary/ tertiary health system contact points.	-Non-community settings/institutions.
Type of Evidence Source	Publication type	-Peer-reviewed journal articles.	-Editorials. -Conference papers. -Unpublished theses. -Reports. -Study protocol papers. -Studies focused on incidence or prevalence of cancers
Time Frame	Publication period.	-Inception to 18 February 2024.	-Publications outside this time frame.
Language	Language of the publication.	-Studies published in English.	-Studies not published in English.

**Table 2:** The type of cancer screening under review.

Type of Cancer Screening Under Review	Number of Studies/Articles	Cited Literature
Breast	7	[23,24,25,26,27,28,29]
Cervical	6	[30,31,32,33,34,35]
Breast and cervical	4	[36,37,38,39]
Colon	4	[40,41,42,43]
Lung	1	[44]
Breast and colon	1	[45]
Breast, bowel and cervical	1	[46]
Various cancers	13	[47,48,49,50,51,52,53,54,55,56,57,58,59]

**Table 3:** Recommendations to improve cancer screening in the literature.

Recommendation	Subthemes
Improve health system	-Tailored interventions. -Target groups, risk factors, areas of concern. -Physical and mental health system integration. -Address barriers at service user and physician levels. -Revise current care models. Consider implementing co-location of services.
Social determinants of health	-Increase funding. -Understand insurance schemes. -Cultural safety. Address racial/ethnic disparities. Various languages. -Patient navigation programs. -Educate providers and patients.
Improve participation	-Directly ask about screening. -Prompt patients. -Address issues at the screening invitation process. -Reduce stigma around mental illness. -Improve communication. -Support systems.
Research	-Barriers and facilitators of cancer screening. -Interventions and care settings.
Other/ miscellaneous	-Individual, policy, and system level strategies. -Guidelines and policies.

## Data Availability

The original contributions presented in this study are included in the article/Supplementary Materials. Further inquiries can be directed to the corresponding author.

## Supplementary Materials

The following supporting information can be downloaded at https://www.mdpi.com/article/10.3390/ijerph23020216/s1, Supplementary File S1: Table S1. Example output table from MEDLINE database search; Supplementary File S2: Table S2. Study characteristics summary; Supplementary File S3: Table S3. MMAT v.18 quality rating for qualitative studies, Table S4. MMAT v.18 quality rating for quantitative randomized controlled trials, Table S5. MMAT v.18 quality rating for quantitative non-randomized trials, Table S6. MMAT v.18 quality rating for quantitative descriptive studies, Table S7. MMAT v.18 quality rating for mixed methods studies, Table S8. Peer-reviewed literature—CASP quality rating of systematic reviews; Supplementary File S4: Table S9. Summary of findings: Barriers, facilitators, recommendations; Supplementary File S5: Table S10. Thematic table of recommendations; Supplementary File S6: Table S11. Thematic table of facilitators; Supplementary File S7: Table S12. Thematic table of recommendations.

## Abbreviations

The following abbreviations are used in this manuscript:

CASP	Critical Appraisal Skills Programme
CMHC	Community Mental Health Clinic
FIT	Faecal immunochemical test
gFOBT	Guaiac faecal occult blood test
GP	General practitioner
HPV	Human papilloma virus
MMAT	Mixed Methods Appraisal Tool
NHB	Non-Hispanic Black
NHO	Non-Hispanic other
NHW	Non-Hispanic White
NSW	New South Wales
PCP	Primary care provider
RACGP	Royal Australian College of General Practitioners
RCT	Randomized controlled trial
USA	United States of America
UK	United Kingdom
v.18	Version 2018
