# Supplementary material for: A Descriptive Thematic Review of Barriers, Facilitators, and Recommendations for Cancer Screening Uptake Among Community Dwelling Adults with Mental Ill-Health
Source: Int J Environ Res Public Health. 2026 Feb 9;23(2):216. doi: 10.3390/ijerph23020216 (PMC12940195; doi:10.3390/ijerph23020216)
Supplement: Supplementary file 1 [file ijerph-23-00216-s001.zip › ijerph-4072675-supplementary.pdf]

Supplementary File S1

Table S1. Example output table from MEDLINE database search.

Database(s): Ovid MEDLINE(R) and Epub Ahead of Print, In-Process, In-Data-Review & Other Non-Indexed Citations, Daily and Versions 1946 to September 09, 2022
Search Strategy:

#	Searches	Results
1	("mental health" OR "mental illness").mp. [mp=title, book title, abstract, original title, name of substance word, subject heading word, floating sub-heading word, keyword heading word, organism supplementary concept word, protocol supplementary concept word, rare disease supplementary concept word, unique identifier, synonyms]	265838
2	(miss* OR "under screened" OR reduced OR "less likely" OR "influences").mp. [mp=title, book title, abstract, original title, name of substance word, subject heading word, floating sub-heading word, keyword heading word, organism supplementary concept word, protocol supplementary concept word, rare disease supplementary concept word, unique identifier, synonyms]	2381028
3	("cancer screening" OR "cancer prevention" OR "cancer detection").mp. [mp=title, book title, abstract, original title, name of substance word, subject heading word, floating sub-heading word, keyword heading word, organism supplementary concept word, protocol supplementary concept word, rare disease supplementary concept word, unique identifier, synonyms]	66943
4	1 and 2 and 3	66

Supplementary File S2

Table S2. Study Characteristics Summary

Surname of First Author, Year, Country	Aims/purpose	Participant Samples	Methods/design	Findings/conclusions	Limitations
#1) Abuelo (2020), UK [47]	Evaluate the impact of a patient navigation program on colorectal cancer screening in patients with mental illness and/or substance use disorder<br>Evaluate the feasibility and effectiveness of patient navigation for patients with mental illness and/or substance use disorder who receive care in a community health centre and are overdue for colorectal cancer screening	-251 patients<br>-50-74yrs with mental illness and/or substance use disorder due for colorectal cancer screening	-pilot randomized nonblinded controlled trial	-intervention led to more colorectal cancer screening<br>-navigation program was more effective in younger males	-generalizability<br>-difficulty with navigators reaching screening patients<br>-training focus changed during the study<br>-effectiveness of screening (majority of practices not using FIT screening)
#2) Aggarwal (2013), USA [36]	Review current evidence on breast and cervical cancer screening disparities in women with mental illness	-varying sample sizes (26 to 335294)<br>-various population samples	-systematic literature review<br>-prospective and retrospective studies<br>-cross-sectional and qualitative studies<br>-small and large databases	-differences in breast and cervical cancer screenings	
#3) Baillargeon (2011), USA [41]	Evaluate the extent to which preexisting mental disorders influence diagnosis, treatment, and survival in older adults with colon cancer	-67+yrs with diagnosed colon cancer<br>-n= 80,670	-retrospective cohort study	-poorer cancer outcomes for people with mental disorders	-sample population (Medicare coverage)<br>-physicians can underrecognize mental illness

					-participants can under report mental illness<br>-generalizability<br>-selection bias and confounding<br>-socioeconomic status limited by census
#4)<br>Barley (2013), England [48]	Determine the effectiveness of interventions targeted at adults with SMI, or their carers or health professionals, and aimed at increasing the uptake of cancer screening tests for which the adults with SMI are eligible	-18+ yrs with SMI and eligible for cancer screening	-relevant RCT<br>-Cochrane Schizophrenia Group's Trials Register	-no RCT evidence to promote uptake of cancer screening in people with SMI<br>-no studies met the inclusion criteria	
#5)<br>Barley (2016), England [49]	Determine the effectiveness of interventions targeted at adults with SMI, or their carers or health professionals, and aimed at increasing the uptake of cancer screening tests for which the adults with SMI are eligible	-18+yrs with SMI and eligible for cancer screening	-RCTs<br>-Cochrane Schizophrenia Group’s Trials Register	-no RCT evidence to promote uptake of cancer screening in people with SMI<br>-no studies met the inclusion criteria	
#6)<br>Borrull-Guardeno (2019), USA [32]	Determine the cervical cancer screening rates in women with SMI	-women 25-65yo in psychiatric hospital during 2016	-descriptive cross-sectional study<br>-consecutive sampling	-highest screening rates in 35-44yo, lowest in 55-65yo<br>-screened less than general population	-small sample size
#7)<br>Cespedes (2020), Spain [45]	Compare the severity of breast and colorectal cancers at diagnosis in people with and without mental disorders<br>Assess possible differences according to specific groups of mental disorders (schizophrenia spectrum disorders and mood disorders), as well as the influence of age upon the delay in	-111 oncology patients (75 had breast cancer and 6 had colorectal cancer)<br>-patients were identified from a study with the Health Department	-observational, retrospective, case-control study<br>-stratified random allocation for controls**	-more likely to be diagnosed with advanced cancer if they have SMI<br>-more advanced diagnosis in older people with mental illness<br>-common factors for discrepancies	-small sample size<br>-generalizability<br>-unable to evaluate role of hereditary cancers

	detecting breast cancer or colorectal cancer				
#8)<br>Clifton (2016), UK [46]	Identify barriers and facilitators for breast, cervical and bowel cancer screening uptake by people with mental illness in order to inform interventions to promote equal access	-85ppl interviewed; eligible for either breast, cervical, or bowel cancer screening<br>-45 service users, 11 screening professionals, 29 mental health professionals<br>-ages ranged from 26-73yrs	-theoretical domains framework<br>-snowball sampling	-key themes related to uptake of screening programs<br>-barriers and facilitators; these vary depending on the type of screening<br>-need a collaborative approach	-less men in sample study<br>-limited participant mention of bowel cancer screening<br>-sample population size<br>-influenced interpretation<br>-validity of participant groups<br>-type of mental illness can affect screening uptake
#9)<br>Domino (2015), USA [50]	Examine whether enrolment in a primary care medical home alters the patterns of care for Medicaid enrollees with SMI	-18+yrs with minimum of 2 outpatient visits or 1 inpatient visit and diagnosis of bipolar, schizophrenia, or major depression<br>-N=272149	-retrospective secondary data analysis	-enrolment improves mental health care use<br>-significant association for preventive services (ex: screening) in major depressive disorder	-Medicaid claims data<br>-measure of psychiatric severity not available<br>-medication adherence based on filled claims<br>-selection bias<br>-confounder (mental health service reforms)
#10)<br>Eriksson (2019), Sweden [31]	Explore whether women with psychiatric diagnoses participate in cervical cancer screening programs to a lesser extent than women on average, and whether there are disparities between psychiatric diagnostic groups based on grades of severity	-23-60yrs with minimum of two times being diagnosed with ICD-10 codes, registered at primary care or psychiatric care, and residents of Västra Götaland<br>-65292 women	-3 registers	-less screening participation if they get psychiatric specialist care<br>-diagnosis of psychosis have lowest screening participation	-scale not validated for grouping psychiatric diagnoses<br>-underestimation of data
#11)	Examine the current evidence from existing literature on the risk of	-varying populations	-literature review	-lower screening rates in people with SMI	-narrative review<br>-need complete searches

Grassi (2023), USA [51]	developing cancer and the course of the disease among people with SMI, specifically schizophrenia/schizophrenic spectrum disorders and mood (bipolar, major depression) disorders			-factors associated with lower screening rates	
#12) Happell (2012), USA [52]	-Examine whether disparities in preventative health care for cancer and infectious disease exist for individuals living with SMI, potential contributors to health care inequalities, and to make recommendations for the mental health nursing profession based on these findings	-varying populations	-narrative review approach<br>-population-based cohort studies or administrative claims data	-mixed evidence: most show less preventive services for people with SMI however others show appropriate level of care	-bias (non-systematic approach)<br>-limited comparability<br>-generalizability<br>-reliability (retrospective analysis)<br>-frequency of people with SMI seeking physical health care
#13) Harder (2018), Denmark [30]	Describe non-participants in the Danish cervical cancer screening program, including potential differences in socio-demographic characteristic between active and passive non-participants<br>Assess the role of socio-demographic factors, reproductive history, and mental and physical health for passive non-participation in cervical cancer screening in Denmark	-n=543325<br>-study population = 476670<br>-23-49yr old and invited for cervical cancer screening<br><br>Groups<br>-screening participants n=402984 (registered in databank and in 4yr follow-up period)<br>-non-participants (n=10251) actively unsubscribed<br>-passive non-participants (n=63435) were either group	-nationwide register-based cohort study	-younger women tend to actively unsubscribe<br>-all age groups had similar amounts of passive non-participation<br>-lower income led to greater risk of passive non-participation<br>-women with no prior screening history had greater chance of being passive non-participant<br>-risk factors for passive non-participation	-limited analysis of health care provider and organization characteristics<br>-measure of obesity<br>-measurement of health<br>-limited information on influences of screening participation

#14) Impelido (2023), Australia [33]	Describe overall and age-specific cervical cancer screening rates in mental health service users in NSW	-20-69yrs (n=114022) mental service user -other NSW women (n=2110127) -NSW resident	-linked data (National Cervical Screening Program)	-less screening participation for mental health service users -screening varies depending on age	-impact of COVID-19 on cancer screening and participation -missing diagnoses limited subgroup analysis -generalizability -not aimed at high risk subgroups -underestimate disadvantage in screening gaps -lack reliable data on cultural background
#15) Irwin (2014), USA [53]	Summarizes known disparities in cancer prevention, diagnosis, treatment, and end-of-life care among individuals with schizophrenia		-review article	-greater risk of lung cancer in people with schizophrenia -higher chance of dying from cancer if schizophrenic -people with schizophrenia are less likely to have current screenings	
#16) Irwin (2019), USA [44]	Establish the prevalence of older adults with schizophrenia who meet eligibility criteria for lung cancer screening, investigate patient lung cancer risk perceptions, and examine patient-reported PCP access and clinician delivery of smoking cessation interventions	-112 adults in community mental health clinic (CMHC) on clozapine -schizophrenia or schizoaffective disorder -regular CMHC visits -18+yrs (18 and older)	-cross-sectional survey -medical records -descriptive statistics	-older age is more likely to underestimate risk of lung cancer -underestimated risk of developing ling cancer -PCP delivery of smoking cessation intervention is mediocre -smoking cessation and views vary based on age	-study sample -generalizability -questionnaire not validated

#17) Kilbourne (2011), USA [54]	Determine whether patients with serious mental illness receiving care in Veterans Affairs mental health programs with collocated general medical clinics were more likely to receive adequate medical care than patients in programs without collocated clinics based on a nationally representative sample	-minimum of 1 SMI diagnosis	-cross-sectional study	-collocated clinics were more likely to do screening<br>-generally better quality of care at collocated clinics	-misclassification bias<br>-limited ability to draw relations about quality of care<br>-limited outcome data<br>-generalizability
#18) Kodl (2010), USA [55]	Examine the association between mental health diagnoses and colorectal cancer screening, and to test the hypothesis that the frequency of outpatient visits confounds this relationship	-855 veterans aged 50-75yrs sampled from 29882 veterans with at least 1 primary care visit at "Minneapolis VA Medical Center"	-observational study<br>-retrospective cohort	-veterans less likely to be screened	-generalizability<br>-diffiuclt to determine the effect of medical record prompts and screening<br>-only included screening done/paid by veterans affairs
#19) Koroukian (2012), USA [26]	Determine whether disparities exist in receipt of screening mammography between women with and without mental illness enrolled in Medicaid, a program with documented potential to reduce healthcare disparities	-50-64yr<br>-female<br>-enrolled in Ohio's Medicaid during 2002-2008 (n=130088)	-Ohio Medicaid enrollment and claims files	-women with mental illness had lower chance of getting screened<br>-more likely to be screened with Medicaid enrollment	-claims data might not be done
#20) Lambeth (2023), Australia [24]	Describe the rate of BreastScreen participation in women aged 50 to 74 who have had contact with specialist mental health services in New South Wales (NSW), Australia, and compare this to rates in the general population after adjusting for age, socioeconomic status and rurality	-50-74yrs when screened<br>-NSW residents<br>-minimum of 1 contact with mental health service in the previous 2yrs or from 2015-2018	-population-wide data linkage	-less screening participation in mental health service users<br>-screening gaps<br>-more screening in women with persistent or SMI compared to other mental health service users<br>-barriers	-biased socioeconomic gradient<br>-underestimate screening gap in advantaged areas<br>-generalizability<br>-sub-group definitions

#21) Lawrence (2014), Australia [56]	Discuss the complexities associated with providing medical care for people with comorbid psychiatric disorders and the difficulties faced by people with mental illness and the people who provide them with medical care		-record linkage systems	-mixed results about screening in mentally ill<br>-likely to be under screened if have mental illness	
#22) Linz (2022), USA [25]	Identify barriers and facilitators toward breast cancer screening in women diagnosed with SMI	-40+yr women with SMI<br>-treated in community mental health service<br>-15 women interviewed	-interpretive descriptive approach<br>-qualitative data	-breast cancer screening in women with SMI has various barriers and facilitators	-loss of funding<br>-lack quantitative methods
#23) Mitchell (2014), UK [29]	Conduct a systematic review and meta-analysis to establish if women with a mental health condition are less likely to receive mammography screening compared with those without mental ill health	-women with non-organic psychiatric disorder	-comparative studies<br>-systematic review<br>-meta-analysis	-women with mental health conditions had lower mammography screening rates	-heterogeneity in analyses<br>-generalizability<br>-unable to investigate risk factors for low mammography uptake<br>-varying time frame<br>-retrospective histories
#24) Mkuu (2022), USA [34]	Identify barriers that healthcare providers face in providing cervical cancer screening to women with behavioural health conditions<br>-examine provider perspectives on barriers	-4 focus groups of varying size (minimum n=5 to maximum n=10)<br>-26 clinicians	-purposive convenience sampling<br>-thematic analysis<br>-qualitative focus group data from mixed methods study	-main barriers<br>1) behavioral health conditions<br>2) system level<br>3) barriers similar for general population	-limited number of focus groups<br>-purposive sampling<br>-self-selection bias<br>-effect of group dynamics on responses<br>-no insurance demographics
#25) Murphy (2021), USA [57]	Describe receipt of cancer screening among individuals with versus without SMI and to explore clinicians' options around cancer screening for people with SMI	-cervical cancer screening: 21-64yrs<br>-breast cancer screening: 50-64yrs	-retrospective study<br>-mixed-methods study<br>-semi-structured interviews	-less likely to have cancer screening if have SMI<br>-themes possibly related to lower screening rates	-generalizability<br>-time interval of data analysis<br>-study participants<br>-unable to determine if test was ordered but not

	Examine cancer screening rates in a national sample of commercially insured adults<br>-identify perceived barriers and facilitators to cancer screening for the SMI and general population through interviews with primary care providers (PCPs) and psychiatrists	-colorectal cancer screening: men and women 50-64yrs<br>-prostate cancer screening: men 55-64yrs<br>-SMI			completed or due to other reasons<br>-generalizability
#26)<br>Ouk (2020), Canada [35]	Estimate whether bipolar disorder or schizophrenia is associated with disparities in cervical cancer screening rates<br>-compare the likelihood and frequency of cervical cancer screening in community-dwelling women with and without a history of bipolar disorder or schizophrenia from the general population in Ontario	-19-69yr women in community<br>-Ontario resident<br>-119948 diagnosed with bipolar disorder or schizophrenia in 2003-2012<br>-1245457 women in total	-retrospective population-based matched case-cohort study	-exposure was less likely to receive screening and was also screened less often	-generalizability<br>-unable to account for various sociodemographic factors at individual level<br>-validity of algorithm<br>-no sole estimates for major depressive disorder<br>-population sample may not include all eligible
#27)<br>Sara (2023), Australia [27]	Compare the degree of spread of newly diagnosed invasive breast cancers in women with and without recent MH service contact, after adjusting for participation in breast cancer screening and for other potential confounders	-women 50-74yrs with invasive breast cancer diagnosis and in NSW Cancer Registry<br>-residents of NSW	-population-wide linkage study<br>-descriptive statistics	-lower screening participation in mental health service users<br>-more advanced cancer related to low screening participation<br>-reduced participation doesn't account for main reason mental health service users have greater risk of advanced cancer	-study population size<br>-limited ability to analyze precision of cancer severity description<br>-three-level measure of screening history<br>-risk factors for screening participation not included in data set<br>-lack individual data for assessing socioeconomic index

#28) Siantz (2017), USA [40]	Examine mental illness as a risk factor for nonadherence to colorectal cancer screening guidelines	-people aged 50+ of California (N=15,535) -doctor recommended them to be screened in the previous 5yrs	-cross-sectional observational study	-couldn't determine a relationship between adherence to colorectal cancer screening and mental illness -other factors for nonadherence	-self-reported data -type of mental illness can affect adherence -timing issue -selection bias -not generalizable -limited variables
#29) Thomas (2018), USA [28]	Examine mammogram screening rates among women with SMI by using socioecological framework	-females 48-67yrs, prescription claim for antipsychotic medication, use specialty mental health care, and enrolled in Medi-Cal	-retrospective cohort study (N=14651)	-disparity in screening rates between people with mental illness and national average -screening rates vary based on psychiatric diagnosis	-timing (1yr of screening data) -age of study population includes <50yr
#30) Thomsen (2023), Netherlands [43]	Examine the extent to which people with mental disorders participate in organised colorectal cancer screening	-history of mental disorder and invited to screening -Danish residents 50-74yrs	-population-based cohort study	-less participation for people with mental disorders -more positive faecal immunochemical test results, less adherence/incomplete colonoscopies in people with mental disorders	
#31) Tsai (2023), USA [42]	Examine colorectal cancer screening utilization among non-Hispanic White, non-Hispanic Black (NHB), non-Hispanic other (NHO)/Hispanic cancer survivors -whether experiencing poor physical and/or mental health affects colorectal cancer screening utilization in breast	-1325697 respondents -18+ yrs	-cross-sectional survey	-less colorectal cancer screening in NHO/Hispanic survivors -less colorectal cancer screening seen in psychiatric disorders	-overestimation of colorectal cancer screening -cross-sectional analysis -limited ability to analyze barriers for population -interpretation bias

	and prostate cancers across different racial/ethnic groups			-no study has compared screening and mental health in various races/ethnicities	-retrospective data
#32) Ukhanova (2020), USA [58]	Identify multimorbidity combinations associated with low receipt of preventive services	-301,665 patients<br>-19+ (19 and older) with 2 or more ambulatory visits	-retrospective cohort study	-less preventative measures, such as cancer screenings, in mental health only multimorbidity group	-limitations with enrollment continuity<br>-generalizability<br>-individual preferences that influence access to preventive care
#33) Vigod (2011), Canada [39]	Investigate the relationship between depression and screening for breast and cervical cancer	-N=4042 cervical cancer screening. 18-67yrs<br>-N-1403 breast cancer screening. 50-68yrs<br>-Ontario residents	-cross-sectional survey	-women with depressive symptoms had less mammography screening<br>-women with depressive symptoms are less likely to get pap test if aged 40-70 compared to others	-generalizability<br>-not meant to determine mechanisms for reduced cancer screening<br>-unsure if depression was present before follow-up
#34) Werneke (2006), UK [23]	Determine uptake rates of breast screening services in women with mental illness in inner London Assess the impact of age, social deprivation, severity of illness, and screening experience	-933 psychiatric patients and 44195 women with no mental health issue<br>-50-64yrs<br>-registered at South London hospital or Maudsley hospital	-cross-sectional data linkage study	-psychiatric patients and reference group were as likely to attend screening overall<br>-patients with psychosis or SMI were less likely to attend screening<br>-predictors of non-attendance	-limited ability to further analyze risk factors of non-attendance<br>-accuracy of databases<br>-underestimated proportion of SMI
#35) Woodhead (2016), England [38]	Breast and cancer screening uptake has been found to be lower among women with SMI. This study aims to corroborate these findings in the UK and to identify variation in screening	-SMI patients eligible for breast and cervical screening (n=625 and n=1393) in primary care	-linked population-based data<br>-meta-analysis	-less screening for patients with SMI<br>-factors are associated with decreased screening rates	-generalizability<br>- mammography is not performed in primary care, information may be less complete,

	uptake by illness/treatment factors, and primary care consultation frequency	-those with no SMI (n=106554 and n=25385)<br>-mammography: 50-70yrs<br>-cervical cancer screening: 25-64yrs		-primary care contact influences cervical screening more<br>-increase in primary care contact is associated with increase in mammography screening	particularly for those who consult less often<br>-study population
#36) Yarborough (2018), USA [59]	Compare facilitators and rates of colorectal cancer screening between people with and without mental illnesses	-age eligible, average risk of colorectal cancer, and minimum of 266 days since last gFOBT	-cohort study<br>-secondary analysis	-screening rates of people with mental illness can increase with FIT	-lacking information<br>-observational study and timing<br>-sample population<br>-limited comparability to other studies
#37) Zhang (2020), USA [37]	Explore the association between anxiety and depressive symptoms and breast and cervical cancer screening behaviour in a general population	-21-65yrs eligible for cervical cancer screening<br>-50-74yrs eligible for breast cancer screening<br>-n=3014	-cross-sectional survey	-association between anxiety and chance of missing breast cancer screening<br>-significant association between depression and chance of missing breast/cervical cancer screening<br>-significant association between anxiety and cervical cancer screening<br>-other factors for reduced screenings	-cross-sectional study so hard to determine causation<br>-many participants didn't report income<br>-generalizability to racial/ethnic groups

Supplementary File S3

Table S3. MMAT v.18 quality rating for qualitative studies

Author/date	1.1. Is the qualitative approach appropriate to answer the research question?	1.2. Are the qualitative data collection methods adequate to address the research question?	1.3. Are the findings adequately derived from the data?	1.4. Is the interpretation of results sufficiently substantiated by data?	1.5. Is there coherence between qualitative data sources, collection, analysis and interpretation?	Overall quality*
Lawrence et al. (2014) #21	Y	Y	Y	Y	Y	***
Mkuu et al. (2022) #24	Y	Y	Y	Y	Y	***
Linz & Jerome-D'Emilia, (2022) #22	Y	Y	Y	Y	Y	***

Y = Yes, N = No, C = Can't Tell

*MMAT v.18 quality rating: low = 1 to 2 stars; moderate = 3 stars; moderately high = 4 stars; high = 5 stars [20]

Table S4. MMAT v.18 quality rating for quantitative randomized controlled trials

Author/date	2.1. Is randomization appropriately performed?	2.2. Are the groups comparable at baseline?	2.3. Are there complete outcome data?	2.4. Are outcome assessors blinded to the intervention provided?	2.5 Did the participants adhere to the assigned interventio n?	Overall quality *
Abuelo et al. (2020) #1	Y	Y	Y	N	C	***

Y = Yes, N = No, C = Can't Tell

*MMAT v.18 quality rating: low = 1 to 2 stars; moderate = 3 stars; moderately high = 4 stars; high = 5 stars [20]

Table S5. MMAT v.18 quality rating for quantitative non-randomized trials.

Author/date	3.1. Are the participants representative of the target population?	3.2. Are measurements appropriate regarding both the outcome and intervention (or exposure)?	3.3. Are there complete outcome data?	3.4. Are the confounders accounted for in the design and analysis?	3.5. During the study period, is the intervention administered (or exposure occurred) as intended?	Overall quality*
Domino et al. (2015) #9	Y	Y	Y	Y	Y	****
Ouk et al. (2020) #26	Y	Y	Y	Y	Y	****
Siantz et al. (2017) #28	Y	Y	Y	Y	Y	****
Thomas et al. (2018) #29	Y	Y	Y	Y	Y	****
Zhang et al. (2020) #37	Y	Y	Y	Y	Y	****

Y = Yes, N = No, C = Can't Tell

*MMAT v.18 quality rating: low = 1 to 2 stars; moderate = 3 stars; moderately high = 4 stars; high = 5 stars [20]

Table S6. MMAT v.18 quality rating for quantitative descriptive studies

Author/Date	*4.1.* Is the sampling strategy relevant to address the research question?	*4.2.* Is the sample representative of the target population?	*4.3.* Are the measurements appropriate?	*4.4.* Is the risk of nonresponse bias low?	*4.5.* Is the statistical analysis appropriate to answer the research question?	*Overall quality**
Baillargeon et al. (2011) #3	Y	Y	Y	Y	Y	****
Borrull-Guardeno et al. (2019) #6	Y	Y	Y	Y	Y	***
Cespedes et al. (2020) #7	Y	Y	Y	Y	Y	****
Eriksson et al. (2019) #10	Y	Y	Y	Y	Y	****
Harder et al. (2018) #13	Y	Y	Y	Y	Y	*****
Impelido et al. (2023) #14	Y	Y	Y	N**	Y	****
Irwin et al. (2019) #16	Y	Y	Y	Y	Y	***
Kilbourne et al. (2011) #17	Y	Y	Y	N***	Y	****
Kodl et al. (2010) #18	Y	Y	Y	Y	Y	****
Koroukian et al. (2012) #19	Y	Y	Y	Y	Y	***
Lambeth et al. (2023) #20	Y	Y	Y	Y	Y	****
Sara et al. (2023) #27	Y	Y	Y	Y	Y	*****
Thomsen et al. (2023) #30	Y	Y	Y	Y	Y	****
Tsai et al. (2023) #31	Y	Y	Y	Y	Y	***
Ukhanova et al. (2020) #32	Y	Y	Y	Y	Y	***
Vigod et al. (2011) #33	Y	Y	Y	N****	Y	***
Werneke et al. (2006) #34	Y	Y	Y	Y	Y	***
Woodhead et al. (2016) #35	Y	Y	Y	Y	Y	****
Yarborough et al. (2018) #36	Y	Y	Y	Y	Y	****

Y = Yes, N = No, C = Can't Tell *MMAT v.18 quality rating: low = 1 to 2 stars; moderate = 3 stars; moderately high = 4 stars; high = 5 stars [20]; ** 40.3% of service users participated in National Cervical Screening Program for the past 2 years; *** 81% survey completion rate; **** 77% person level response rate*

Table S7. MMAT v.18 quality rating for mixed methods studies.

Author/Date	5.1. Is there an adequate rationale for using a mixed methods design to address the research question?	5.2. Are the different components of the study effectively integrated to answer the research question?	5.3. Are the outputs of the integration of qualitative and quantitative components adequately interpreted?	5.4. Are divergences and inconsistencies between quantitative and qualitative results adequately addressed?	5.5. Do the different components of the study adhere to the quality criteria of each tradition of the methods involved?	Overall quality*
Clifton et al. (2016) #8	Y	Y	Y	Y	Y	***
Murphy et al. (2021) #25	Y	Y	Y	Y	Y	****

Y = Yes, N = No, C = Can’t Tell

*MMAT v.18 quality rating: low = 1 to 2 stars; moderate = 3 stars; moderately high = 4 stars; high = 5 stars [20]

Table S8. Peer-reviewed literature - CASP quality rating of systematic, scoping or narrative reviews

Author/Date	Clearly focused question	Right type of papers	All the important, relevant studies included	Quality of included studies assessed	Reasonable to combine the results	Overall results	Preciseness of results	Applicability of results to local population	All important outcomes are considered	Benefits worth harms & costs
Aggarwal et al. (2013) #2	Y	Y	Y	Y	Y	Y	C	C	Y	Y
Barley et al. (2013) #4	Y	Y	Y	Y	C	Y	C	C	Y	Y
Barley et al. (2016) #5	Y	Y	Y	Y	C	Y	C	C	Y	Y
Grassi et al. (2023) #11	Y	Y	Y	N	Y	Y	C	C	Y	Y
Happell et al., (2012) #12	Y	Y	Y	C	Y	Y	C	C	Y	Y
Irwin et al., (2014) #15	Y	Y	Y	N	Y	Y	Y	Y	Y	Y
Mitchell et al., (2014) #23	Y	Y	Y	Y	Y	Y	C	C	Y	Y

Y = Yes, N = No, C = Can't Tell

Supplementary File S4

Table S9. *Summary of Findings: Barriers, Facilitators, Recommendations*

Surname of First Author, Year, Country	Aims/purpose	Findings (Barriers/Facilitators/Recommendations)
#1) Abuelo (2020), UK	Evaluate the impact of a patient navigation program on colorectal cancer screening in patients with mental illness and/or substance use disorder<br>Evaluate the feasibility and effectiveness of patient navigators for patients with mental illness and/or substance use disorder who receive care in a community health centre and are overdue for colorectal cancer screening	<u>Recommendations</u><br>-navigation program<br>-"larger studies in diverse care settings"
#2) Aggarwal (2013), USA	Review current evidence on breast and cervical cancer screening disparities in women with mental illness	<u>Barriers</u><br>-age, race/ethnicity, education/health literacy<br>-no primary care provider<br>-medical comorbidities<br><u>Mixed (Barrier/Facilitator)</u><br>-specific diagnosis<br>-provider gender<br>-primary care provider<br><u>Barriers in Schizophrenia</u><br>-under-reporting<br>-denial, symptom minimization and tolerance, poor insight<br><u>Barriers in Depressive Symptoms</u><br>-neglect screening due to self-neglect<br>-helplessness, sense of lack of control<br><u>Facilitators</u><br>-trust<br>-compliance

		-social support<br>-primary care provider<br><u>Recommendations</u><br>-use "global functional indicator that measured the overall impact of mental illness" for better categorization of screening influences
#3) Baillargeon (2011), USA	Evaluate the extent to which preexisting mental disorders influence diagnosis, treatment, and survival in older adults with colon cancer	<u>Barriers</u><br>-psychosocial and economic barriers to medical care<br>-complex healthcare needs<br>-incomplete evaluation<br>-ability to access routine care<br>-behavioural factors (ex: smoking)<br><u>Recommendations</u><br>-more research on behavioural, socioeconomic, and biological influences<br>-research on communication, treatment adherence, and medical decision-making
#4) Barley (2013), England	Determine the effectiveness of interventions targeted at adults with severe mental illness, or their carers or health professionals, and aimed at increasing the uptake of cancer screening tests for which the adults with severe mental illness are eligible	<u>Barriers</u><br>-low income, increasing age, lack of transport<br>-embarrassment<br>-lack of reminders<br>-lack of familiar care providers<br><u>Recommendations</u><br>-"Interventions to encourage cancer screening uptake in people with severe mental illness"<br>-"large, multi-centre RCTs" to test interventions<br>-research to tackle barriers<br>-"research whether there are barriers specific to people with SMI"
#5) Barley (2016), England	Determine the effectiveness of interventions targeted at adults with severe mental illness, or their carers or health professionals, and aimed at Increase uptake of cancer screening tests for which the adults with severe mental illness are eligible	<u>Barriers</u><br>-low income, increasing age, lack of transport<br>-embarrassment<br>-lack of reminders<br>-lack of familiar care providers<br><u>Recommendations</u><br>-"Interventions to encourage cancer screening uptake in people with severe mental illness"<br>-"large, multi-centre RCTs" to test interventions<br>-research to tackle barriers<br>-"research whether there are barriers are specific to people with severe mental illness"

#6) Borrull-Guardeno (2019), USA	Determine the cervical cancer screening rates in women with severe mental disorders	<u>Barriers</u><br>-age<br>-severe mental disorders<br><u>Recommendations</u><br>-cancer prevention promotion via mental health nurses (ex: diet, physical activity, smoking cessation, screening options, appointment booking assistance)
#7) Cespedes (2020), Spain	Compare the severity of breast and colorectal cancers at diagnosis in people with and without mental disorders<br>Assess possible differences according to specific groups of mental disorders (schizophrenia spectrum disorders and mood disorders), as well as the influence of age upon the delay in detecting breast cancer or colorectal cancer	<u>Barriers</u><br>-age<br>-diagnostic overshadowing<br>-previous mental disorder (affects cancer care)<br><u>Recommendations</u><br>-"the adoption of organizational strategies described in the Quality in the Continuum for Cancer Care framework: leadership at multiple levels, delivery system design, clinical information systems, and patient self-management support."<br>-educate nurses (mental health, screening, oncology)<br>-interventions to motivate and educate patients (ex: self-exam techniques)<br>-"assign the mental health nurse an appointment management and companion role for screening participation"<br>-community: informative campaigns<br>-make severe mental disorders "a risk factor for inequity in access to cancer care"
#8) Clifton (2016), UK	Identify barriers and facilitators for breast, cervical and bowel cancer screening uptake by people with mental illness in order to inform interventions to promote equal access	<u>Themes</u><br>-knowledge of screening<br>-knowledge of attitudes regarding mental illness<br>-health service delivery factors<br>-service user beliefs and concerns<br>-practicalities for service users<br><u>Professionals Facilitators</u><br>-knowledge (lack of knowledge, not prioritizing screening, emotional/physical barriers to screening)<br>-skills<br>-social influences<br>-professional role and identity<br>-emotion, behavioural regulation and environmental context and resources<br><u>Service User Themes</u>

-knowledge (expectations/need/processing ability)
-skills
-social influences
-memory, attention and decision processes
-beliefs about consequences
-motivation
-emotion, behavioural regulation and environmental context and resources
Recommendations
-individual, policy and service-delivery level
-integration amongst professionals
Theme: Knowledge of screening programmes and processes
Constituent barriers
SU: Not knowing what to expect or what to do; unsure of need for screening; difficult to process information
SP: Communication skills training not available to all MHP: Lack of knowledge of programme and/or procedures; promotion of screening not prioritised; lack of physical health expertise
Constituent facilitators
SU: Wanting to be informed; understanding the benefits of screening; feeling health conscious; encouragement
MHP: Health promotion seen as their role; aware that SU are at risk of cancer; understanding emotional and practical barriers to screening uptake for SU
Theme: Knowledge of, and attitudes towards mental illness
Constituent barriers
SU: Lack of understanding of mental illness in screening professionals; made to feel like a burden on health service; stigma of mental illness
SP: Lack of knowledge of severe mental illness; find complex patients difficult
MHP: Stigma of mental illness (among others)
Constituent facilitators
SU: Staff being understanding; staff knowledge of mental illness
SP: Understanding of emotional and practical barriers to screening uptake for SU; staff motivated to encourage screening for all groups; importance of good communication skills recognised; confidence to
screen anyone associated with good communication skills
Theme: Health service delivery factors

		<u>Constituent barriers</u><br>SU: Screening environment aggravates mental health symptoms; staff can be rushed; staff can be rough; exclusion from GP registers<br>SP: Lack of time; no means of knowing patient needs in advance; computer systems not linked<br>MHP: Lack of structured behaviour change approach /collaboration between healthcare services; no-one has clear responsibility to promote screening; patient's mental state; lack of resources<br><u>Constituent facilitators</u><br>SU: Continuity of care<br>SP: Practice nurses can access patients' records; reactive measures in place if advance notice of need is given<br>MHP: Diagnostic overshadowing known to be a problem; willingness to promote screening; cancer screening promotion included in routine health promotion<br><u>Theme:</u> Service users' beliefs and concerns<br><u>Constituent barriers</u><br>SU: Additional burden; mental health symptoms reduce motivation for self-care; past negative experience; embarrassment; traumatising; fear of bad news; poor relationship with GP; diagnostic overshadowing<br><u>Constituent facilitators</u><br>SU: Feeling health conscious; being anxious to avoid further health problems; physical symptoms (e.g. finding a lump); past positive experience; good relationship with GP; good relationship with practice nurse<br>SP/MHP: Awareness of some of these difficulties<br><u>Theme:</u> Practicalities for service users<br><u>Constituent barriers</u><br>SU: Appointment booking; transport difficulties; difficulty remembering appointments; difficulty leaving the house due to mental health problems; taking time off<br><u>Constituent facilitators</u><br>SU: Familiar location; reminders<br>SP/MHP: Awareness of some of these difficulties"
#9)<br>Domino<br>(2015), USA	Examine whether enrolment in a primary care medical home alters the patterns of care for Medicaid enrollees with severe mental illness	<u>Barriers/Facilitators</u> (depending on type of mental illness)<br>-less integration in treatment of psychotic conditions<br>-specialty mental health visits<br>-physician knowledge

		<u>Recommendations</u><br>-more knowledge about the impact of medical homes on patient care disorder.
#10) Eriksson (2019), Sweden	Explore whether women with psychiatric diagnoses participate in cervical cancer screening programs to a lesser extent than women on average, and whether there are disparities between psychiatric diagnostic groups based on grades of severity	<u>Barriers</u><br>-"impersonal and anonymous nature" of cervical screening<br><u>Facilitators</u><br>-continuity of care (pap test)<br><u>Recommendations</u><br>-distribute information via invitations, materials, films, or orally<br>-yearly counselling about "somatic status"<br>-better encouragement by psychiatric specialists for cervical screening
#11) Grassi (2023), USA	Examine the current evidence from existing literature on the risk of developing cancer and the course of the disease among people with severe mental illness, specifically schizophrenia/ schizophrenic spectrum disorders and mood (bipolar, major depression) disorders	<u>Recommendations</u><br>-reduce stigma<br>-promote healthy behaviour/lifestyle<br>-multi-disciplinary screening programs<br>-control for age and sex
#12) Happell (2012), USA	Examine whether disparities in preventative health care for cancer and infectious disease exist for individuals living with severe mental illnesss, potential contributors to health care inequalities, and to make recommendations for the mental health nursing profession based on these findings	<u>Barriers</u><br>-reduced access<br>-systemic issues<br>-lack of motivation<br>-cognitive difficulties<br><u>Recommendations</u><br>-identify barriers to access<br>-comparative studies with prospective design<br>-integration<br>-educate mental health nurses on "high prevalence of risk behaviors and chronic disorders"<br>-mental health nurses, consumer organizations, and consumers need to collaboratively advocate for government funding
#13) Harder (2018), Denmark	Describe non-participants in the Danish cervical cancer screening program, including potential differences in socio-demographic characteristic between active and passive non-participants	<u>Barriers</u><br>-low income, education level<br>-history of intoxicant abuse, schizophrenia, or other psychoses<br>-socio-demographic characteristic<br>-reproductive history

	-assess the role of socio-demographic factors, reproductive history, and mental and physical health for passive non-participation in cervical cancer screening in Denmark	-mental and physical health<br>-origin other than Denmark (ex: language barrier)<br>-smoke while pregnant<br>-history of induced abortion<br><u>Barriers</u> for Obese Women<br>-fear of embarrassment<br>-lectures about weight loss<br>-inadequate equipment for body size<br><u>Recommendations</u><br>-information in multiple languages<br>-tailor to various cultural backgrounds<br>-option to choose gender of examiner<br>-"plan initiatives to increase screening participation"<br>-community intervention trials in minority groups
#14) Impelido (2023), Australia	Describe overall and age-specific cervical cancer screening rates in mental health service users in NSW	<u>Barriers</u><br>-socioeconomic disadvantage<br>-rural, age, access, cost, education<br>-stigma<br>-health literacy<br>-trauma<br>-nature of cervical screening<br><u>Facilitators</u><br>-private insurance<br>-self-collection for cervical cancer<br><u>Recommendations</u><br>-more research to find the benefit of screening chances, groups at risk, and improvements to health system
#15) Irwin (2014), USA	Summarize known disparities in cancer prevention, diagnosis, treatment, and end-of-life care among individuals with schizophrenia	<u>Facilitators</u><br>-community-based cancer navigators<br><u>Recommendations</u><br>-patient level: interventions for smoking cessation and weight loss<br>-provider level: communication between mental health and oncology professionals<br>-systems level: clinical guidelines and "delineation of responsibility"<br>-integration

		-target patients lost to follow-up<br>-need research about smoking cessation in schizophrenia<br>-study new models of care<br>-involve people with schizophrenia who aren't receiving mental health treatment to extend the population being studied
#16) Irwin (2019), USA	Establish the prevalence of older adults with schizophrenia who meet eligibility criteria for lung cancer screening, investigate patient lung cancer risk perceptions, and examine patient-reported PCP access and clinician delivery of smoking cessation interventions	<u>Barriers</u><br>-cognitive impairments<br>-patient level: underestimation of cancer risk, cognitive deficits, negative symptoms, social isolation<br>-clinician level: education on tobacco cessation and lung cancer screening, safety of tobacco cessation medications, role definition<br>-system level: integration<br>-who's role to provide tobacco cessation<br><u>Recommendations</u><br>-integration<br>-targeted interventions for lung cancer screening and tobacco cessation<br>-interventions should vary based on age<br>-"guidelines should account for differences in risk perceptions by smoking status"<br>-reassess the screening age in patients with schizophrenia<br>-education about risk for former smokers<br>-clinicians should build rapport and share the decision making<br>-investigate barriers at the physician level<br>-research on the effect of CMHC-based interventions
#17) Kilbourne (2011), USA	Determine whether patients with serious mental illness receiving care in Veterans Affairs (VA) mental health programs with collocated general medical clinics were more likely to receive adequate medical care than patients in programs without collocated clinics based on a nationally representative sample	<u>Barriers</u><br>-separate payment and insurance structures<br>-provider networks<br>-limited availability of primary care providers<br><u>Recommendations</u><br>-other strategies for patients with co-occurring chronic medical conditions<br>-chronic care models or "system-redesign models" in collocated services
#18) Kodl (2010), USA	Examine the association between mental health diagnoses and colorectal cancer screening, and to test the hypothesis that the frequency of outpatient visits confounds this relationship	<u>Barriers</u><br>-psychosis had a negative association to screening<br>-diagnosis of mental health conditionand screening had a negative association<br><u>Facilitators</u>

		-more visits to mental health facility<br>-trust in provider<br>-access<br>-1 or more mental health diagnoses<br>-PTSD diagnosis
#19) Koroukian (2012), USA	Determine whether disparities exist in receipt of screening mammography between women with and without mental illness enrolled in Medicaid, a program with documented potential to reduce healthcare disparities	<u>Facilitators</u><br>-"increased length of Medicaid enrollment improved uptake of screening mammography"<br><u>Recommendations</u><br>-research on barriers and facilitators
#20) Lambeth (2023), Australia	Describe the rate of BreastScreen participation in women aged 50 to 74 who have had contact with specialist mental health services in New South Wales (NSW), Australia, and compare this to rates in the general population after adjusting for age, socioeconomic status and rurality	<u>Barriers</u><br>-age, socioeconomic status<br>-rurality (less clear relationship)<br>-physical accessibility<br>-provider attitudes regarding mental illness<br>-concerns about stigma or embarrassment<br>-lack of integration<br><u>Barriers</u> for Breast and Cervical Cancer Screening<br>-shame, stigma<br>-lack of integration<br>-past experience of sexual trauma<br><u>Recommendations</u><br>-universal screening programs need "additional strategies to reach disadvantaged and high-risk groups" and they need to prioritize women using mental health services<br>-cultural safety<br>-co-design services with communities
#21) Lawrence (2014), Australia	Discuss the complexities associated with providing medical care for people with comorbid psychiatric disorders and the difficulties faced by people with mental illness and the people who provide them with medical care	<u>Barriers</u><br>-no ongoing physical healthcare<br>-stigma<br>-cost/insurance<br>-cognitive impairments (ex: less motivation, concentration, assertiveness, etc)<br>-reduced access<br>-impact of other comorbidities<br><u>Facilitators</u>

		-contact with primary care physician<br>-peer support workers<br><u>Recommendations</u><br>-"research into ways to improve health service delivery to people with comorbid mental health problems"<br>-holistic care model (ex: multidisciplinary team and co-location)
#22)<br>Linz (2022), USA	Identify barriers and facilitators toward breast cancer screening in women diagnosed with severe mental illness	<u>Barriers</u><br>-psychiatric symptoms (trauma, fear, distrust, stigma, racism, not how they were raised, not a priority)<br>-gender of clinician<br>-socioeconomic status<br><u>Facilitators</u><br>-good health care experience<br>-easy (ex: transport, reminders, incentives)<br>-integrated support<br>-self-care<br><u>Recommendations</u><br>-health education<br>-interventions for risk factors at individual level (ex: smoking)<br>-intervention targeted towards fragmented care
#23)<br>Mitchell (2014), UK	Conduct a systematic review and meta-analysis to establish if women with a mental health condition are less likely to receive mammography screening compared with those without mental ill health	<u>Barriers</u><br>-distress<br>-low rates of help-seeking<br>-cognitive impairment<br>-primary care provider doesn't "take the physical healthcare complaints of people with mental illness seriously"<br><u>Facilitators</u><br>-primary care provider (trust and support too)<br><u>Recommendations</u><br>-more attention to invitation process (ex: follow-up call)<br>-better communication<br>-educate and support patients

#24) Mkuu (2022), USA	Identify barriers that healthcare providers face in providing cervical cancer screening to women with behavioral health conditions<br>Examine provider perspectives on barriers	Themes specific to behavioural health conditions cervical screening<br>1) behavioural health conditions related barriers<br>-history of trauma<br>-pre-existing conditions<br>2) system level barriers related to behavioural health are<br>-stigma and discrimination<br>-lack of access<br>-out of pocket expenses<br>3) similar barriers to general population<br>-time limitation<br><u>Barriers</u> shared by general population<br>-lack of integration, no reminders<br>-no insurance<br>-challenges communicating with patients<br>Associations-anxiety, fear, shame<br><u>Barriers</u> from Healthcare providers<br>-lack of integration<br>-lack of follow-up<br>-lack of identifying women due for screening<br>-lack of resources to support those with limited access<br><u>Recommendations</u><br>-trauma informed care<br>-help patients navigate and comprehend their insurance plans
#25) Murphy (2021), USA	Describe receipt of cancer screening among individuals with versus without SMI and to explore clinicians' perceptions around cancer screening for people with severe mental illness<br>-examine cancer screening rates in a national sample of commercially insured adults<br>Identify perceived barriers and facilitators to cancer screening for the severe mental illness and general population through interviews with primary care providers (PCPs) and psychiatrists	<u>Clinician Barriers</u> to Screening<br>-access to care (insurance, PCP, stigma)<br>-available support<br>-prioritization of other medical issues<br>-communication<br>-patient concerns (ex: trauma, procedure, fear)<br><u>Recommendations</u><br>-"strategies should account for psychosocial factors (e.g. trauma history, social support), barriers to care (e.g. transportation, insurance), and neurocognitive impairment"<br>-interventions like simple educational material<br>-integration

#26) Ouk (2020), Canada	Estimate whether bipolar disorder or schizophrenia is associated with disparities in cervical cancer screening rates<br>Compare the likelihood and frequency of cervical cancer screening in community-dwelling women with and without a history of bipolar disorder or schizophrenia from the general population in Ontario	Barriers<br>-no family physician<br>-mental health conditions (for cervical cancer screening)<br>-attitude of physicians, stigma<br>-not prioritizing prevention<br>Facilitators<br>-targeted invitations<br>-phone counselling<br>-address financial barriers<br>-continuity of care<br>Recommendations<br>-research other barriers<br>-audits of family physician practices<br>-other health professionals encourage screening at family physician practice
#27) Sara (2023), USA	Compare the degree of spread of newly diagnosed invasive breast cancers in women with and without recent MH service contact, after adjusting for participation in breast cancer screening and for other potential confounders<br>Build data to understand and reduce premature mortality in people using NSW mental health services	Barriers<br>-distress (mixed results)<br>Recommendations<br>-health system and relationship barriers (cost, physical accessibility, provider attitudes, knowledge about mental illness, concerns of stigma, past negative experiences of health care, lack of integration<br>-explore cancer diagnosis (via screening or alternative way)<br>-study of contributing mechanisms and interactions
#28) Siantz (2017), USA	Examine mental illness as a risk factor for nonadherence to colorectal cancer screening guidelines	Barriers to screening adherence<br>-delaying care<br>-low health literacy<br>-females who received screening<br>-age 50-65yrs<br>Facilitators<br>-insurance<br>-poor self-rated health<br>Recommendations<br>-more outreach

#29) Thomas (2018), USA	Examine mammogram screening rates among women with severe mental illness by using socioecological framework	<u>Recommendations</u><br>-more outreach<br>-more communication<br>-integration (ex: sharing mammogram results with practitioners, more outreach, communicating results in special sessions)<br>-methods at a clinical, systemic, and policy level
#30) Thomsen (2023), Netherlands	Examine extent to which people with mental disorders participate in organized colorectal cancer screening	<u>Recommendations</u><br>-GP or mental health facility to provide support
#31) Tsai (2023), USA	Examine colorectal cancer screening utilization among non-Hispanic White, non-Hispanic Black (NHB), non-Hispanic other (NHO)/Hispanic cancer survivors -whether experiencing poor physical and/or mental health affects colorectal cancer screening utilization in breast and prostate cancers across different racial/ethnic groups	<u>Barriers</u><br>-prefer non-general practice lead preventive care<br>-less willing to seek screening<br>-financial barrier for follow-up<br>-poor physical and/or mental health<br>-racial/ethnic differences<br><u>Recommendations</u><br>-consider racial/ethnic disparities<br>-culturally tailored patient navigation programs<br>-research on cultural-specific barriers
#32) Ukhanova (2020), USA	Identify multimorbidity combinations associated with low receipt of preventive services	<u>Barriers</u><br>-prioritization of other health demands/delayed care<br>-mental illness (ex: decline screening)<br>-fragmented care in the United States<br><u>Recommendations</u><br>-interventions<br>-integration (ex: of behavioural health providers into primary care)<br>-primary care provider education regarding screening and bias
#33) Vigod (2011), Canada	Investigate the relationship between depression and screening for breast and cervical cancer	<u>Barriers</u><br>-depression and/or psychological distress<br><u>Recommendations</u><br>-research if screening improves when depressive symptoms are treated<br>-primary care provider can “optimize screening compliance”<br>-have primary care providers ask about “compliance with preventive screening”

#34) Werneke (2006), UK	Determine uptake rates of breast screening services in women with mental illness in inner London<br>Assess the impact of age, social deprivation, severity of illness, and screening experience	<u>Barriers</u><br>-enhanced care status<br>-psychosis diagnosis<br>-history of 2+ admissions<br>-age, ethnicity<br>-social deprivation <u>Recommendations</u><br>-more flexible invitation and appointment systems<br>-staff to explain and encourage patients
#35) Woodhead (2016), England	Breast and cancer screening uptake has been found to be lower among women with serious mental illness. This study aims to corroborate these findings in the UK and to identify variation in screening uptake by illness/treatment factors, and primary care consultation frequency	<u>Facilitators</u><br>-primary care contact (cervical cancer and breast cancer)<br>-incentives (cervical screening) <u>Barriers</u><br>-depot/severe mental illness/not help-seeking<br>-unfamiliar location/staff (mammography) Recommendations<br>-policies that target increased screening
#36) Yarborough (2018), USA	Compare facilitators and rates of colorectal cancer screening between people with and without mental illnesses	<u>Facilitators</u><br>-reminders<br>-at-home test kits<br>-FIT compared to gFOBT<br>-FIT more convenient
#37) Zhang (2020), USA	Explore the association between anxiety and depressive symptoms and breast and cervical cancer screening behavior in a general population	Barriers associated with higher chance of missing Pap test<br>-older age, never been married, lower education level<br>-no insurance<br>-smoking<br>-anxiety<br>-delaying care<br>Barriers associated with higher chance of missing mammogram<br>-older age<br>-no insurance<br>-smoking<br>-rural<br>-delaying care

	Recommendations<br>-"targeted programs and messages for individuals with anxiety and depression"<br>-research with regard to association between anxiety and cervical cancer screening

Supplementary File S5

Table S10. Thematic table of recommendations.

Recommendation Themes	Author, (Year)
**1) Health System**	
More outreach	Siantz (2017)<br>Thomas (2018)<br>Irwin (2014) -involve people with schizophrenia who aren't receiving mental health treatment to extend the population being studied
Integration	Clifton (2016)<br>Ukhanova (2020)<br>Happell (2012)<br>Murphy (2021)<br>Irwin (2019) -integration and shared decision making (with patients too)<br>Sara (2023) -health system and relationship barrier<br>Thomas (2018)<br>Irwin (2014)
Interventions	Cespedes (2020) -to motivate<br>Barley (2013) -to encourage screening uptake; RCTs to test interventions<br>Barley (2016) -to encourage screening uptake; RCTs to test interventions<br>Ukhanova (2020)<br>Harder (2018) –"community intervention trials in minority groups"<br>Murphy (2021) -intervention like educational material<br>Irwin (2019) -interventions for lung cancer screening and tobacco cessation; based on age; research on effect of CMHC-based interventions<br>Irwin (2014) -intervention for smoking cessation and weight loss<br>Linz (2022) -intervention for risk factors at individual level (ex: smoking); interventions targeted towards fragmented care<br>Grassi (2023) -multi-disciplinary screening programs
Targeted programs/consider culture	Zhang (2020) -in ppl with anxiety and depression<br>Tsai (2023) –"culturally tailored patient navigation programs"<br>Abuelo (2020) -navigation program -helpful for minorities. Call patients, educate, remove barriers like transport/insurance/language barriers<br>Lambeth (2023) -universal screening programs targeted towards disadvantaged ppl and females<br>Sara (2023) -targeted approach<br>Lambeth (2023) -cultural safety<br>Harder (2018) -tailor to various cultural backgrounds (ex: languages)<br>Irwin (2014)- target homeless (> people with schizophrenia)<br>Irwin (2019) -targeted methods for people with schizophrenia (better awareness of screening)
Identify barriers to access	Happell (2012) -barriers to access<br>Irwin (2019) -barriers at physician level<br>Cespedes (2020) -make severe mental disorders a risk factor for access<br>Sara (2023) -health system and relationship barriers
Care models	Lawrence (2014) -holistic care model (co-location)<br>Irwin, (2014) -study new models of care; implement care plans<br>Kilbourne, (2011) -chronic care models in co-located services

	Cespedes, (2020) -strategies from Quality in the Continuum for Cancer Care framework
**2) Social Determinants of Health**	
Advocate for more funding	Happell (2012) -mental health nurses, consumer organizations, and consumers advocate for government funding to improve nurses’ ability to improve patient health (more education, resources, etc)
Consider racial/ethnic disparities	Tsai (2023) -racial/ethnic disparities
Educate	Cespedes (2020) -educate nurses and patients; informative community campaigns
	Ukhanova (2020) -primary care provider education re screening bias
	Happell (2012) -educate mental health nurses
	Murphy (2021) -interventions like simple educational material
	Irwin (2019) -educate risk for former smokers; educate professionals on screening
	Mitchell (2014) -educate patients
	Linz (2022) -health education
	Werneke (2006) -staff to explain and encourage patients
	Grassi (2023) -promote healthy behaviour/lifestyle
	Borrull-Guardeno (2019) -cancer prevention promotion via mental health nurses (ex: diet, physical activity, smoking cessation, screening options, appointment booking assistance)
	Eriksson (2019) -yearly counselling about “somatic status”
**3) Compliance/Participation**	
Improve compliance/ participation	A) compliance/participation
	Vigod (2011) -primary care provider “optimize screening compliance”; inquire about compliance
	Ouk (2020) -other health professionals encourage screening at practice
	Irwin (2014) -target patients lost to follow-up
	Harder (2018) –initiatives to increase participation
	Eriksson (2019) -better encouragement by psychiatric specialists for cervical screening
	Woodhead (2016) -characterize based on factors other than diagnosis for patients with SMI
	B) easier invitation and appointments
	Werneke (2006) -easier invitations and appointments
	Mitchell (2014) -more attention to invitation process (ex: follow-up call)
	C) Choose examiner gender
	Harder (2018)
Reduce stigma	Grassi (2023)
	Sara (2023)
Distribution of information	Harder (2018) -in multiple languages
	Eriksson, (2019) -via invitations, materials, films, or orally
Enhanced communication	Thomas (2018) -communicating results in special sessions
	Mitchell (2014) -better communication
	Irwin (2014) -provider level: between MH/oncology professionals
Support	Thomsen (2023) -by GP or mental health facility
	Mitchell (2014) -support patients
	Cespedes (2020) -patient self-management support
	Lawrence (2014) -primary care provider and peer support workers
**4) Recommended Solutions**	

Audits of family physician practices	Ouk (2020)
Explore cancer diagnosis	Sara (2023) -via screening or alternative way<br>If women living with mental health conditions face other barriers in access to primary care -may be more likely to have cancers diagnosed through free screening programs vs clinical examination by a physician. Further analysis could explore alternative measures, distinguish between cancers diagnosed at screening and "interval" cancers diagnosed through other means.
Rapport and shared decision making	Irwin (2019) -by clinicians
Reassess screening age	Irwin (2019) -in patients with schizophrenia
Understand insurance	Mkuu (2022) -help patients navigate, comprehend their insurance plans
Trauma informed care	Mkuu (2022)
Research	Barley (2013) -to tackle barriers; whether specific to people with SMI<br>Zhang (2020) -research with regard to association between anxiety and cervical cancer screening<br>Baillargeon (2011) -research on behavioral, socioeconomic, and biological influences; research on communication, treatment adherence, and medical decision-making<br>Barley (2016) -research to tackle barriers; research on if barriers are specific to people with SMI<br>Tsai (2023) -on cultural-specific barriers<br>Irwin (2019) -on effect of CMHC-based interventions Irwin (2014) -about smoking cessation in schizophrenia<br>Vigod (2011) - screening improves when depressive symptoms are treated Impelido (2023) -benefit of screening chances, groups at risk, and improvements to health system<br>Ouk (2020) -on barriers<br>Koroukian (2012) -barrier and facilitators<br>Lawrence (2014) -improving delivery of health services in people with comorbid mental health issues<br>Happell (2012) -comparative studies with prospective design<br>Abuelo (2020) –"larger studies in diverse care settings" to determine most effective navigator<br>Sara (2023) -study of contributing mechanisms and interactions<br>Domino (2015) -more knowledge about the impact of medical homes on patient care disorder
**5) Other**	
Other strategies	Kilbourne (2011) -patients with co-occurring chronic medical conditions<br>Murphy (2021) -account for psychosocial factors, barriers to care, and neurocognitive impairment
Individual, policy and service-delivery level	Clifton (2016)<br>Cespedes (2020) -delivery system design. Also "companion role for screening participation" -assigned roles for mental health nurse<br>Lambeth (2023) -co-design services with communities<br>Thomas (2018) -methods at a clinical, systemic, and policy level<br>Irwin (2014) -systems level: clinical guidelines/delineation responsibility Woodhead (2016) -policies that target increased screening in SMI<br>Irwin (2019) –"guidelines should account for differences in risk perceptions by smoking status"

Supplementary File S6

Table S11. Thematic table of facilitators

Themes for Facilitators	Author, (Year)
**1) Social Determinants**	
Insurance/Finances	Siantz (2017) -insurance<br>Impelido (2023) -private insurance<br>Koroukian (2012) -increased Medicaid enrolment for mammography screening<br>Ouk (2020) -address financial barriers
Transport is easy	Linz (2022)
Familiar location	Clifton (2016) -service user
**2) Increasing Uptake in the Health System**	
Knowledge	Clifton (2016) -professional facilitator and service user facilitator
Professional role and identity	Clifton (2016) -professionals: mental health
Participation	Yarborough (2018) -phone reminders. Colorectal cancer screening<br>Linz (2022) - easy (ex: reminders)<br>Clifton (2016) -SU reminders<br>Woodhead (2016) -incentives. cervical cancer screening *unsure about impact on likelihood of screening<br>Linz (2022) -easy (ex: incentives)
Test/convenience	Yarborough (2018) -colorectal cancer screening at home kit; FIT compared to gFOBT; FIT more convenient<br>Impelido (2023) -self-collection for cervical cancer
Community-based cancer navigators	Irwin (2014)
Integrated	Linz (2022)<br>Domino (2015) -less integration in treatment of psychotic conditions is barrier/facilitator depending on type of mental illness<br>Kilbourne (2011) – co-located clinics has better quality of care
Targeted invitations	Ouk (2020)
Phone counselling	Ouk (2020)
**3) Trust**	
Trust	Aggarwal (2013)<br>Mitchell (2014)
Provider gender	Aggarwal (2013) -mixed barrier/facilitator
Compliance	Aggarwal (2013)
Positive Experience	Linz (2022) -good health care experience<br>Clifton (2016) -service user past positive experience; good relationship with GP; good relationship with practice nurse
**4) Support**	
Support	Aggarwal (2013) -social support<br>Mitchell (2014) -primary care provider support<br>Linz (2022) -support<br>Lawrence (2014) -peer-support workers

Primary care provider	Aggarwal (2013) -mixed barrier/facilitator<br>Mitchell (2014)<br>Woodhead (2016) -more contact with primary care showed higher screening rates (cervical cancer and breast cancer) through reminders<br>Lawrence (2014) -contact with primary care physician
Continuity of care (under PCP)	Eriksson (2019) -pap test<br>Ouk, (2020)<br>Clifton (2016) -service user<br>Eriksson, (2019) -for pap test<br>Irwin, (2014)
**5) Self-Care**	
Self-care	Linz (2022)
Physical symptoms	Clifton (2016) -service user (ex: finding lump)
Self-rated health	Siantz (2017) -poor self-rated health<br>Clifton (2016) -feeling health conscious for service user
Diagnosis and mental health	Domino (2015) -specialty mental health visits. barrier/facilitator depending on type of mental illness<br>Aggarwal (2013) -specific diagnosis. mixed barrier/facilitator<br>Clifton (2016) -anxious to avoid further health problems. Service user<br>Lambeth (2023)-more severe mental illness<br>Harder (2018) -prior diagnosis of cancer makes women more conscious of need for screening

Supplementary File S7

Table S12. Thematic table of recommendations

Recommendation Themes	Author, (Year)
**1) Health System**	
More outreach	Siantz (2017)<br>Thomas (2018)<br>Irwin (2014) -involve people with schizophrenia who aren't receiving mental health treatment to extend the population being studied
Integration	Clifton (2016)<br>Ukhanova (2020)<br>Happell (2012)<br>Murphy (2021)<br>Irwin (2019) -integration and shared decision making (with patients too)<br>Sara (2023) -health system and relationship barrier<br>Thomas (2018)<br>Irwin (2014)
Interventions	Cespedes (2020) -to motivate<br>Barley (2013) -to encourage screening uptake; RCTs to test interventions<br>Barley (2016) -to encourage screening uptake; RCTs to test interventions<br>Ukhanova (2020)<br>Harder (2018) –"community intervention trials in minority groups"<br>Murphy (2021) -intervention like educational material<br>Irwin (2019) -interventions for lung cancer screening and tobacco cessation; based on age; research on effect of CMHC-based interventions<br>Irwin (2014) -intervention for smoking cessation and weight loss at patient level<br>Linz (2022) -intervention for risk factors at individual level (ex: smoking); interventions targeted towards fragmented care<br>Grassi (2023) -multi-disciplinary screening programs
Targeted programs/consider culture	Zhang (2020) -in ppl with anxiety and depression<br>Tsai (2023) –"culturally tailored patient navigation programs"<br>Abuelo (2020) -navigation program -helpful for minorities. They call patients, educate, remove barriers like transport/insurance/language barriers<br>Lambeth (2023) -universal screening programs targeted towards disadvantaged ppl and females<br>Sara (2023) -targeted approach<br>Lambeth (2023) -cultural safety<br>Harder (2018) -tailor to various cultural backgrounds (ex: languages)<br>Irwin (2014)- target homeless (increase amount of people with schizophrenia)<br>Irwin (2019) -targeted methods for people with schizophrenia (better awareness of screening)
Identify barriers to access	Happell (2012) -barriers to access<br>Irwin (2019) -barriers at physician level<br>Cespedes (2020) -make severe mental disorders a risk factor for access<br>Sara (2023) -health system and relationship barriers

Care models	Lawrence (2014) -holistic care model (co-location)<br>Irwin (2014) -study new models of care; implement care plans<br>Kilbourne (2011) -chronic care models in co-located services<br>Cespedes (2020) -strategies from Quality in the Continuum for Cancer Care framework
**2) Social Determinants of Health**	
Advocate for more funding	Happell (2012) -mental health nurses, consumer organizations, and consumers advocate for government funding to improve nurse's ability to improve patient health (more education, resources, etc)
Consider racial/ethnic disparities	Tsai, (2023) -racial/ethnic disparities
Educate	Cespedes (2020) -educate nurses and patients; informative community campaigns<br>Ukhanova (2020) -primary care provider education regarding screening and bias<br>Happell (2012) -educate mental health nurses<br>Murphy (2021) -interventions like simple educational material<br>Irwin (2019) -educate risk for former smokers; educate professionals on screening<br>Mitchell (2014) -educate patients<br>Linz (2022) -health education<br>Werneke (2006) -staff to explain and encourage patients<br>Grassi (2023) -promote healthy behaviour/lifestyle<br>Borrull-Guardeno (2019) -cancer prevention promotion via mental health nurses (ex: diet, physical activity, smoking cessation, screening options, appointment booking assistance)<br>Eriksson (2019) -yearly counselling about "somatic status"
**3) Compliance/Participation**	
Improve compliance/participation	A) compliance/participation<br>-Vigod (2011) -primary care provider "optimize screening compliance"; inquire about compliance<br>Ouk (2020) -other health professionals encourage screening at family physician practice<br>Irwin (2014) -target patients lost to follow-up<br>Harder (2018) –"initiatives to increase participation"<br>Eriksson (2019) -better encouragement by psychiatric specialists for cervical screening<br>Woodhead (2016) -characterise based on factors other than diagnosis for patients with SMI<br>B) easier invitation and appointments<br>Werneke (2006) -easier invitations and appointments<br>Mitchell (2014) -more attention to invitation process (ex: follow-up call)<br>C) Choose examiner gender<br>Harder (2018)
Reduce stigma	Grassi (2023)<br>Sara (2023)
Distribution of information	Harder (2018) -in multiple languages<br>Eriksson (2019) -via invitations, materials, films, or orally

Enhanced communication	Thomas (2018) -communicating results in special sessions<br>Mitchell (2014) -better communication<br>Irwin (2014) -provider level: btwn mental health and oncology professionals
Support	Thomsen (2023) -by GP or mental health facility<br>Mitchell (2014) -support patients<br>Cespedes (2020) -patient self-management support<br>Lawrence (2014) -primary care provider and peer support workers
**4) Recommended Solutions**	
Audits of family physician practices	Ouk (2020)
Explore cancer diagnosis	Sara (2023) -via screening or alternative way<br>If women living with mental health conditions face other barriers in access to primary care, they may be more likely to have cancers diagnosed through free screening programs rather than through clinical examination by a physician. Further analysis could explore alternative measures or distinguish between cancers diagnosed at screening and "interval" cancers diagnosed through other means.
Rapport and shared decision making	Irwin (2019) -by clinicians
Reassess screening age	Irwin (2019) -in patients with schizophrenia
Understand insurance	Mkuu (2022) -help patients navigate and comprehend their insurance plans
Trauma informed care	Mkuu (2022)
Research	Barley (2013) -to tackle barriers; whether there's barriers specific to people with severe mental illness<br>Zhang (2020) -research with regard to association btwn anxiety and cervical cancer screening<br>Baillargeon (2011) -research on behavioural, socioeconomic, and biological influences; research on communication, treatment adherence, and medical decision-making<br>Barley (2016) -research to tackle barriers; research on if barriers are specific to people with SMI<br>Tsai (2023) -on cultural-specific barriers<br>Irwin (2019) -on effect of CMHC-based interventions<br>Irwin (2014) -about smoking cessation in schizophrenia<br>Vigod (2011) -if screening improves when depressive symptoms are treated<br>Impelido (2023) -benefit of screening chances, groups at risk, and improvements to health system<br>Ouk (2020) -on barriers<br>Koroukian (2012) -barrier and facilitators<br>Lawrence (2014) -improving delivery of health services in people with comorbid mental health issues<br>Happell (2012) -comparative studies with prospective design<br>Abuelo (2020) –"larger studies in diverse care settings" to determine most effective navigator<br>Sara (2023) -study of contributing mechanisms and interactions

	Domino (2015) -more knowledge about the impact of medical homes on patient care disorder
**5) Other**	
Other strategies	Kilbourne (2011) -for patients with co-occurring chronic medical conditions<br>Murphy (2021) -account for psychosocial factors, barriers to care, and neurocognitive impairment
Individual, policy and service-delivery level	Clifton (2016)<br>Cespedes (2020) -delivery system design. Also "companion role for screening participation" -assigned roles for mental health nurse<br>Lambeth (2023) -co-design services with communities<br>Thomas (2018) -methods at a clinical, systemic, and policy level<br>Irwin (2014) -systems level: clinical guidelines and "delineation of responsibility"<br>Woodhead (2016) -policies that target increased screening in severe mental illness<br>Irwin (2019) –"guidelines should account for differences in risk perceptions by smoking status"
